# Metabolites From the Mangrove-Derived Fungus *Cladosporium* sp. HNWSW-1

**DOI:** 10.3389/fchem.2021.773703

**Published:** 2021-12-16

**Authors:** Xi Cao, Lei Guo, Caihong Cai, Fandong Kong, Jingzhe Yuan, Cuijuan Gai, Haofu Dai, Pei Wang, Wenli Mei

**Affiliations:** ^1^ Hainan Key Laboratory for Research and Development of Natural Products From Li Folk Medicine, Hainan Institute for Tropical Agricultural Resources, Institute of Tropical Bioscience and Biotechnology, Chinese Academy of Tropical Agricultural Sciences, Haikou, China; ^2^ Jiangsu Key Laboratory of Marine Bioresources and Environment, Co-Innovation Center of Jiangsu Marine Bio-Industry Technology, Jiangsu Ocean University, Lianyungang, China; ^3^ Key Laboratory of Chemistry and Engineering of Forest Products, State Ethnic Affairs Commission, Guangxi Key Laboratory of Chemistry and Engineering of Forest Products, Guangxi Collaborative Innovation Center for Chemistry and Engineering of Forest Products, School of Chemistry and Chemical Engineering, Guangxi University for Nationalities, Nanning, China

**Keywords:** mangrove-derived fungus, *Cladosporium* sp., metabolites, cytotoxicity, α-glycosidase inhibitor

## Abstract

Two new benzoic acids, cladoslide A (**1**) and cladoslide B (**2**); one new β-carboline derivative, cladospomine (**3**); and one new pyridin-2(1*H*)-one, cladoslide C (**4**), were isolated from the fermentation cultures of the mangrove-derived fungus *Cladosporium* sp. HNWSW-1, along with the previously reported *N*-acetyl-β-oxotryptamine (**5**), (4*S*,5*S*,11*R*)-*iso*-cladospolide B (**6**), (4*S*,5*S*,11*S*)-*iso*-cladospolide B (**7**), and (4*R*,5*S*,11*R*)-*iso*-cladospolide B (**8**). Their structures were elucidated by spectroscopic analysis, Rh_2_(OCOCF_3_)_4_-induced ECD experiments, and Marfey’s method. Compound **1** showed cytotoxicity against the K562 cell line with IC_50_ values of 13.10 ± 0.08 *μ*M. Moreover, compounds **1** and **5** exhibited inhibitory activity against α-glycosidase with IC_50_ values of 0.32 ± 0.01 mM and 0.17 ± 0.01 mM, respectively.

## Introduction

Mangrove-derived fungi are an important resource for structurally and biologically diverse substances for drug discovery, and in recent years, over 100 new molecules derived from mangrove-derived fungi have been discovered every year (Blunt et al., 2018; Carroll et al., 2019; Carroll et al., 2020). The genus *Cladosporium* (Cladosporiaceae) is one of the largest genera of dematiaceous hyphomycetes (Bensch et al., 2015). Many novel bioactive natural products were isolated from *Cladosporium* fungus, such as polyketides (Zhang et al., 2019; Zhu et al., 2018), macrolides (Huang et al., 2019), perylenequinones (Zhang et al., 2020), and indole alkaloids (Peng et al., 2013), which exhibited antimicrobial (Zhang et al., 2019), cytotoxic (Zhu et al., 2018), antiviral (Peng et al., 2013), and quorum-sensing inhibitory activities (Zhang et al., 2020).

As part of our previous research on novel biologically active natural products from mangrove-derived fungi, two novel succinimide-containing derivatives, cladosporitins A and B, have been isolated from the mangrove-derived *Cladosporium* sp. HNWSW-1 (Wang et al., 2019). Our further chemical investigations on this fungus led to the isolation of two new benzoic acids (**1** and **2**), one new β-carboline derivative (**3**), and one new pyridin-2(1*H*)-one (**4**), along with four known compounds (**5**–**8**) (Martínez-Luis et al., 2012; Reddy et al., 2012; Franck et al., 2001) from the EtOAc extract of its fermentation cultures. We describe the isolation, structure elucidation, and biological activities of these compounds in this article.

## Materials and Methods

### General Experimental Procedures

Silica gel (60–80, 200–300 mesh, Qingdao Marine Chemical Co. Ltd.), ODS gel (20–45 m, Fuji Silysia Chemical Co. Ltd.), and Sephadex LH-20 (Merck, Kenilworth, NJ, United States) were used for column chromatography. Optical rotations were measured on a MCP 5100 modular compact polarimeter (Anton Paar, Austria). ECD spectra were recorded on a Bio-Logic Science MOS-500 spectrometer (Biologic, France). UV spectra were measured on a Beckman DU-640 spectrophotometer (Beckman Coulter, Inc., Brea, CA). IR absorptions were obtained on a Nicolet 380 FT-IR instrument (Thermo, Waltham, MA, United States) using KBr pellets. 1D and 2D NMR spectra were recorded on a Bruker AV III spectrometer (Bruker, United States; ^1^H NMR at 500 MHz and ^13^C NMR at 125 MHz for **1**–**3** and **5**; ^1^H NMR at 600 MHz and ^13^C NMR at 150 MHz for **4**, **6**–**8**; and HMBC spectrum for **3**) using TMS as the internal standard. ESIMS and HRESIMS were recorded with amaZon SL (Bruker, United States) or Compact QqTOF (Bruker, United States). Semipreparative HPLC was carried out using an ODS column and 5PFP column (Cosmosil-pack, 10 × 250 mm, 5 μm, 4 ml/min, Nacalai Tesque).

### Fungal Material

The strain of *Cladosporium* sp. HNWSW-1 was isolated from the healthy tree root of *Ceriops tagal*, which was collected from the Dong Zhai Gang Mangrove Reserve in Hainan Province in July 2011 (Wang et al., 2019). The fungus was identified based on the DNA sequences (GenBank access No. MH 535968) of the 18Sr DNA gene (Wang et al., 2019).

### Fermentation and Extraction

*Cladosporium* sp. HNWSW-1 was cultured in PDB (potato liquid media consisting of 200.0 g/L potato, 20.0 g/L glucose, and 1000 ml deionized water) at 28°C and 150 rpm for 72 h. Then, 5 ml seed broth was transferred to 1000 ml Erlenmeyer flasks (60 flasks) each containing rice medium (80.0 g rice, 120.0 ml water, and 120.0 mg tryptophan). The flasks were incubated at room temperature under static conditions for 60 days. The cultures were extracted three times by EtOAc, and the EtOAc solutions were combined and evaporated under reduced pressure to give a dark brown gum (40.0 g). Then, the extracts were dissolved in 90% CH_3_OH, and the solution was extracted three times by petroleum ether. The methanol and petroleum ether solutions were evaporated under reduced pressure. The crude methanol extract (20.0 g) was obtained.

### Purification and Identification

The crude methanol extract (20.0 g) was fractionated into 12 fractions (Fr.1–Fr.12) on a silica gel VLC column eluted with a gradient elution of CH_2_Cl_2_-petroleum ether (0–100%) and MeOH-CH_2_Cl_2_ (0–100%). Fr.6 (2.4 g) was subjected to an Rp-C_18_ silica gel column eluted with a gradient of water-MeOH (10–100%) to give 25 fractions (Fr.6.1–Fr.6.25). Fr.6.6 (535.0 mg) was further chromatographed on the Rp-C_18_ silica gel column using a step gradient with water-MeOH (10–100%) to obtain six fractions (Fr.6.6.1–Fr.6.6.6). Fr.6.6.4 (138.0 mg) was purified by a Sephadex LH-20 column and eluted with MeOH to give three fractions (Fr.6.6.4.1–Fr.6.6.4.3). Fr.6.6.4.1 was submitted to HPLC purification on a 5PFP column eluted with 15% ACN (85% water added to 0.05% trifluoroacetic acid) to yield **7** (11.0 mg, t_*R*_ 10.30 min) and **8** (4.7 mg, t_*R*_ 13.20 min). Fr.6.10 (34.0 mg) was purified by a Sephadex LH-20 column and eluted with MeOH to yield **1** (4.0 mg). Fr.7 (853.1 mg) was submitted to an RP-C_18_ column and eluted with MeOH-water to give 30 fractions (Fr.7.1–Fr.7.30). Fr.7.5 (110.2 mg) was separated by a Sephadex LH-20 column and eluted with MeOH to give four fractions (Fr.7.5.1–Fr.7.5.4). Fr.7.5.1 (32.0 mg) was submitted to HPLC purification on a 5PFP column eluted with 30% MeOH (70% water added to 0.05% trifluoroacetic acid) to yield **6** (12.0 mg, t_*R*_ 23.0 min). Fr.7.12 (45.9 mg) was separated by a Sephadex LH-20 column and eluted with MeOH to give 5 fractions (Fr.7.12.1–Fr.7.12.5). Fr.7.12.5 was submitted to HPLC purification on a 5PFP column eluted with 50% MeOH (50% water added to 0.05% trifluoroacetic acid) to yield **5** (3.8 mg, t_*R*_ 27.0 min). Fr.7.13 (36.4 mg) was also separated by a Sephadex LH-20 column and eluted with MeOH to give eight fractions (Fr.7.13.1–Fr.7.13.8). Fr.7.13.6 was submitted to HPLC purification on a 5PFP column eluted with 40% MeOH (60% water added to 0.05% trifluoroacetic acid) to yield **4** (1.0 mg, t_*R*_ 27.0 min). Fr.7.18 (34.2 mg) was separated by a Sephadex LH-20 column and eluted with MeOH to give five fractions (Fr.7.18.1–Fr.7.18.5). Fr.7.18.3 was submitted to HPLC purification on a 5PFP column eluted with 50% MeOH (50% water added to 0.05% trifluoroacetic acid) to yield **2** (2.0 mg, t_*R*_ 16.9 min). Fr.7.20 was submitted to HPLC purification on a 5PFP column eluted with 30% MeCN (70% water added to 0.05% trifluoroacetic acid) to yield **3** (2.0 mg, t_*R*_ 24.6 min).

### Characterization of Compounds **1–4**

Cladoslide A (**1**): yellow, amorphous powder; [α]^20^_D_ +26.5 (*c* 0.2, MeOH); UV (MeOH) λ_max_ (log ε) 259 (5.39) and 205 (5.42) nm; IR (KBr) ν_max_: 3247, 2941, 1700, 1610, 1261, and 1161 cm^−1^; HRESIMS m/z 305.1006 [M + Na]^+^ (calcd. for C_14_H_18_O_6_Na: 305.0996); ^1^H and ^13^C NMR data (see Table 1).

**TABLE 1:** ^1^H and ^13^C NMR data for **1** and **2** (500 and 125 MHz, *δ* in ppm) in CD_3_OD.

No.	1		2	
	*δ*_C_	*δ*_H_, mult. (*J* in Hz)	*δ*_C_	*δ*_H_, mult. (*J* in Hz)
1	159.4, C	-	159.3, C	-
2	122.2, C	-	122.2, C	-
3	132.9, CH	7.80, d, (2.1)	132.9, CH	7.78, s
4	123.2, C	-	123.3, C	-
5	130.4 CH	7.75, dd, (8.5, 2.1)	130.4, CH	7.73, d, (8.7)
6	118.2, CH	6.79, d, (8.5)	118.1, CH	6.75, d, (8.7)
7	22.7, CH_2_	2.84, m	22.7, CH_2_	2.84, m
8	31.7, CH_2_	1.85, m	31.7, CH_2_	1.84, m
9	77.7, C	-	77.6, C	-
10	36.0, CH_2_	2.01, dt, (14.7, 7.4)	35.9, CH_2_	2.03, dt, (14.3, 7.8)
		1.93, dt, (14.7, 7.9)		1.94, dt, (14.3, 7.9)
11	29.4, CH_2_	2.46, t, (7.9)	29.3, CH_2_	2.50, t, (7.9)
12	177.4, C	-	175.8, C	-
13	24.0, CH_3_	1.30, s	24.0, CH_3_	1.29, s
14	170.1, C	-	170.1, C	-
15	-	-	52.2, OCH_3_	3.65, s

Cladoslide B (**2**): yellow oil; [α]^20^_D_ +6 (*c* 0.04, MeOH); UV (MeOH) λ_max_ (log ε) 259 (5.46) and 205 (5.44) nm; IR (KBr) ν_max_: 3394, 2937, 1722, 1613, 1260, and 1186 cm^−1^; HRESIMS *m/z* 319.1139 [M + Na]^+^ (calcd. for C_15_H_20_O_6_Na: 319.1152); ^1^H and ^13^C NMR data (see Table 1).

Cladospomine (**3**): yellow, amorphous powder; [α]^20^_D_ -4 (*c* 0.2, MeOH); UV (MeOH) λ_max_ (log ε) 362 (4.53), 279 (5.28), and 217 (5.18) nm; IR (KBr) ν_max_: 3341, 2958, 1721, 1653, 1531, 1366, and 1235 cm^−1^; HRESIMS *m*/*z* 392.1227 [M + Na]^+^ (calcd. for C_19_H_19_N_3_O_5_Na: 392.1217); ^1^H and ^13^C NMR data (see Table 2).

**TABLE 2:** ^1^H and ^13^C NMR data for **3** (500 and 125 MHz, *δ* in ppm) in DMSO and **4** (600 and 150 MHz, *δ* in ppm) in CD_3_OD.

No.	3		4	
	*δ*_C_	*δ*_H_, mult. (*J* in Hz)	*δ*_C_	*δ*_H_, mult. (*J* in Hz)
1	142.3, C ^a^	-	-	-
2	-	-	163.3, C ^a^	-
3	131.0, C ^a^	-	117.8, CH	6.40, s
4	119.8, CH	9.14, s	153.2, C	-
5	122.8, CH	8.47, d, (7.8)	109.8, CH	6.31, d, (6.4)
6	121.2, CH	7.35, t, (7.8)	137.4, CH	7.53, d, (6.4)
7	129.9, CH	7.64, t, (7.8)	48.4, CH_2_	4.03, t, (6.8)
8	113.9, CH	7.85, d, (8.1)	24.2, CH_2_	2.03, m
9	-	NH, 12.22, s	30.2, CH_2_	2.36, t, (7.3)
10	136.5, C	-	174.9, C	-
11	132.2, C	-	19.8, CH_3_	2.25, s
12	120.7, C	-	-	-
13	142.7, C	-	-	-
14	165.2, C	-	-	-
15	-	NH, 9.59, s	-	-
16	50.9, CH	4.69, t, (6.40)	-	-
17	174.6, C	-	-	-
18	40.6, CH_2_	1.87, m, 1.77, m	-	-
19	25.0, CH	1.76, m	-	-
20	23.5, CH_3_	0.98, d, (6.0)	-	-
21	21.8, CH_3_	0.96, d, (5.9)	-	-
22	166.2, C	-	-	-

a Assigned from HMBC and HSQC spectra.

Cladoslide C (**4**): yellow oil; UV (MeOH) λ_max_ (log ε) 297 (4.68), 229 (4.72), and 203 (5.08) nm; IR (KBr) ν_max_: 3421, 2955, 1724, 1656, 1570, and 1196 cm^−1^; HRESIMS *m*/*z* 218.0779 [M + Na]^+^ (calcd. for C_10_H_13_NO_3_Na: 218.0788); ^1^H and ^13^C NMR data (see Table 2).

### Rh_2_(OCOCF_3_)_4_-Induced ECD Experiments of **1** and **2**

The samples of compounds **1** and **2** (0.1 mg) were dissolved in a dry solution of the stock [Rh_2_(OCOCF_3_)_4_] complex (1.5 mg) in CH_2_Cl_2_ (1 ml). The first induced ECD spectra of the compounds were recorded immediately after mixing, and their time evolution was monitored until stationary (about 10 min after mixing) (Frelek and Szczepek, 1999). The inherent ECD spectra were subtracted. The absolute configurations of the C-9 tertiary alcohol in **1** and **2** were identified by the observed sign of the E-band at ca. 350 nm in the induced ECD spectra (Gerards and Snatzke, 1990; Frelek and Szczepek, 1999).

### Preparation of FDAA Derivatives of the Acid Hydrolysate of 3 and the Derivatives of Two Authentic Leucine Samples (L- and D-) and Marfey’s Analysis

Compound **3** (1.0 mg, 2.71 *μ*mol) was dissolved in 6 M HCl (1 ml) in a sealed tube, and the mixture was heated at 105°C for 11 h. Then, the solution was cooled and evaporated to dryness. The residue was dissolved in H_2_O (250 μl). Meanwhile, L-Leu and D-Leu were also dissolved in H_2_O (50 mM each), and 50 μl of each solution was treated with 200 μl of 1% FDAA in acetone followed by 1.0 M NaHCO_3_ (40 μl). The reaction was maintained for 1 h at 45°C and then quenched by the addition of 2.0 M HCl (10 μl). The corresponding FDAA derivatives of the hydrolysate of **3**, L- Leu, and D-Leu were analyzed by HPLC on an ODS column maintained at 30 °C using the following programs: solvent A, H_2_O + 0.1% TFA; solvent B, MeCN; linear gradient, 0 min 25% B (75% A), 40 min 60% B (40% A), and 45 min 100% B; UV detection at 340 nm. The retention times for the FDAA derivatives of the hydrolysate of **3**, L-Leu, and D-Leu were 24.28, 24.28, and 28.59 min, respectively (Marfey, 1984).

### Bioassay for Cytotoxicity

The cytotoxic activity of compounds **1**–**3** and **5**–**8** against human cervical cancer cell lines (Hela), human hepatic carcinoma cell lines (BEL-7402), leukemia cell lines (K562), and human gastric cell lines (SGC-7901) was assayed by the MTT method (Mosmann, 1983; Wang et al., 2013). These cell lines were purchased from Shang Hai Cell Bank of Chinese Academy of Sciences. Hela, BEL-7402, K562, and SGC-7901 cell lines were cultured in RPMI-1640 with 10% FBS under a humidified atmosphere of 5% CO_2_ and 95% air at 37°C, and 198 μl of the cell suspension was plated in 96-well microtiter plates. After being incubated for 24 h, 2 μl of the test solutions in DMSO was added to each well and further incubated for 36 h. The MTT solution (20 μl, 5 mg/ml in IPMI-1640 medium) was then added to each well and further incubated for 4 h. Finally, the medium containing MTT (150 μl) was gently replaced by DMSO and pipetted to dissolve any formazan crystals formed. Absorbance was then determined on a Multiskan FC photometric microplate reader (Thermo Fisher Scientific) at 570 nm. Adriamycin was used as the positive control drug.

### Bioassay for α-Glycosidase Inhibitory Activity

α-Glucosidase inhibitory activity of compounds **1**–**3** and **5**-**8** was evaluated according to the literature experimental method (Ma et al., 2014). A mixture including 25 μl of different compounds (final concentrations of 0.0625, 0.125, 0.25, 0.5, and 1.0 mM), 25 μl of α-glucosidase (0.2 U/ml, from baker’s yeast, Sigma), and 175 μl phosphate buffer (pH 6.8) was left to stand for 10 min at room temperature in a 96-well plate, and then 25 μl of 23.2 mM *p*-nitrophenyl *α*-D-glucopyranoside (Sigma-Aldrich) was added and further incubated at 37°C for 15 min. Finally, the absorbance was measured at 405 nm to determine the amount of *p*-nitrophenol cleaved by the enzyme using a Synergy H1 Hybrid Multi-Mode Microplate Reader (BioTek Instruments, Inc.). The control was prepared by adding phosphate buffer instead of the sample in the same way as the test. The blank was prepared by adding phosphate buffer instead of α-glucosidase using the same method. The inhibition rates (%) = [(OD_control_ − OD_control blank_) − (OD_sample_ – OD_sample blank_)]/(OD_control_ − OD_control blank_) × 100%. Acarbose was used as the positive control with an IC_50_ value of 0.72 ± 0.01 mM.

## Results and Discussion

### Identification of Compounds **1**–**4**

**FIGURE 1:**
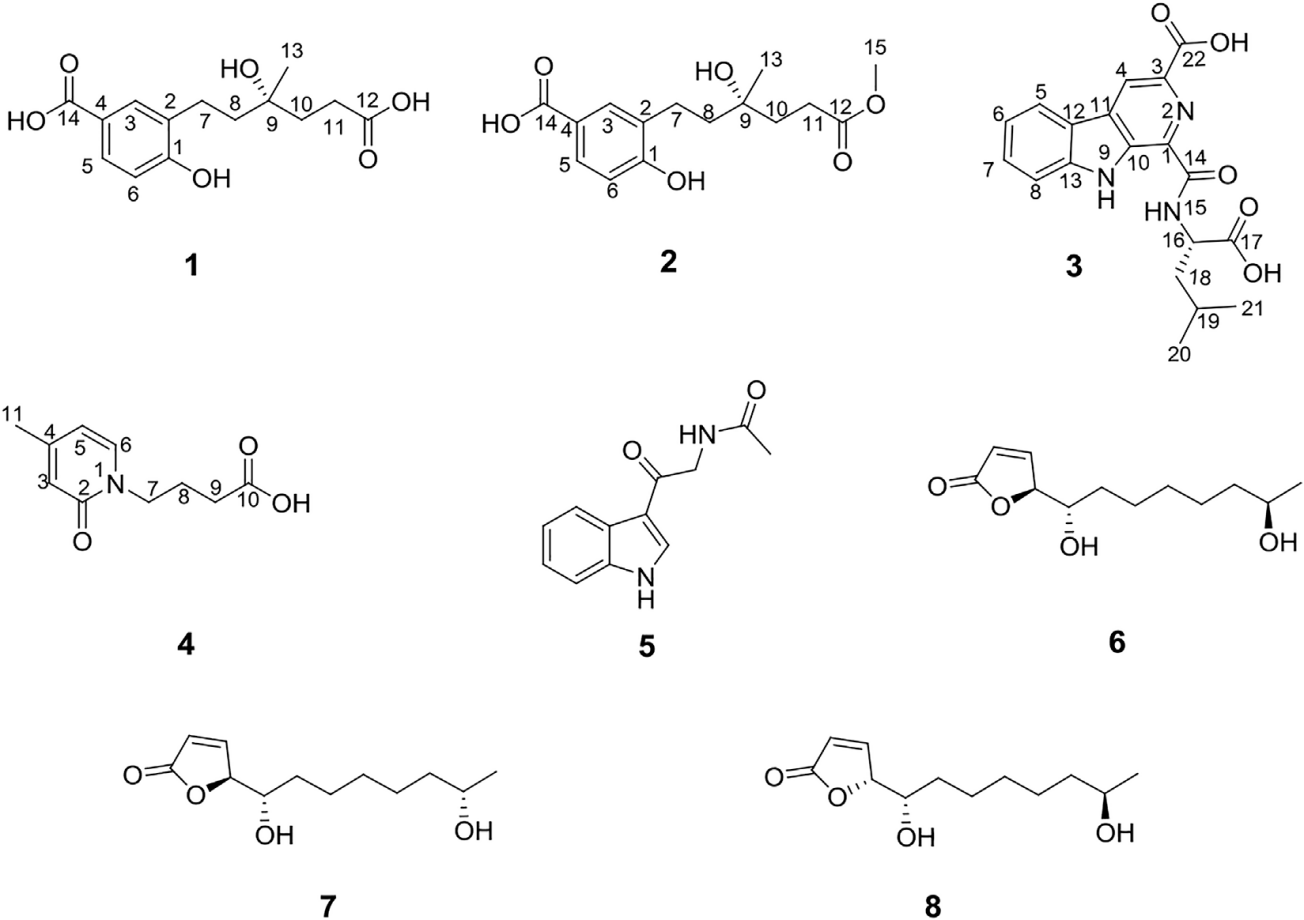
Structures of compounds **1–8**.

**FIGURE 2:**
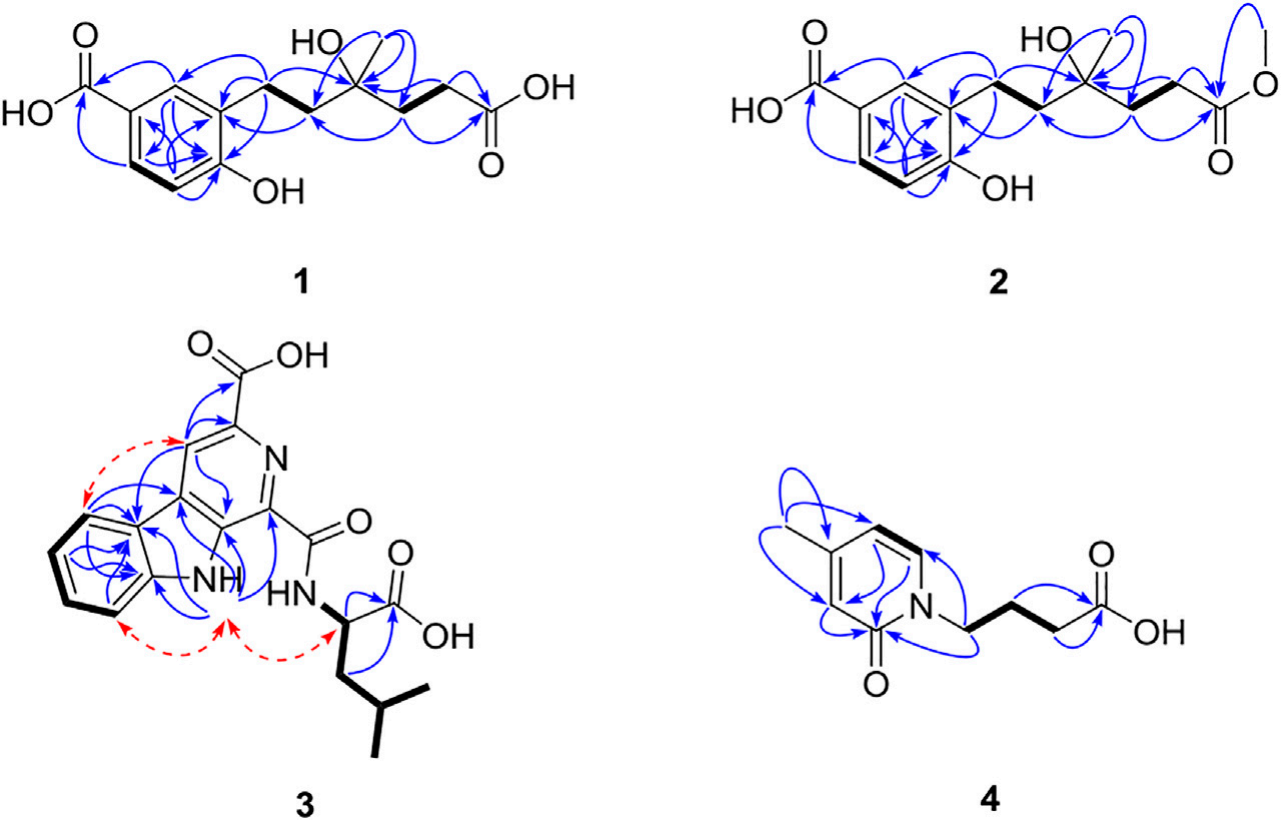
Key HMBC (→), ^1^H–^1^H COSY (▬), and ROESY (← - →) correlations of compounds **1**–**4**.

**FIGURE 3:**
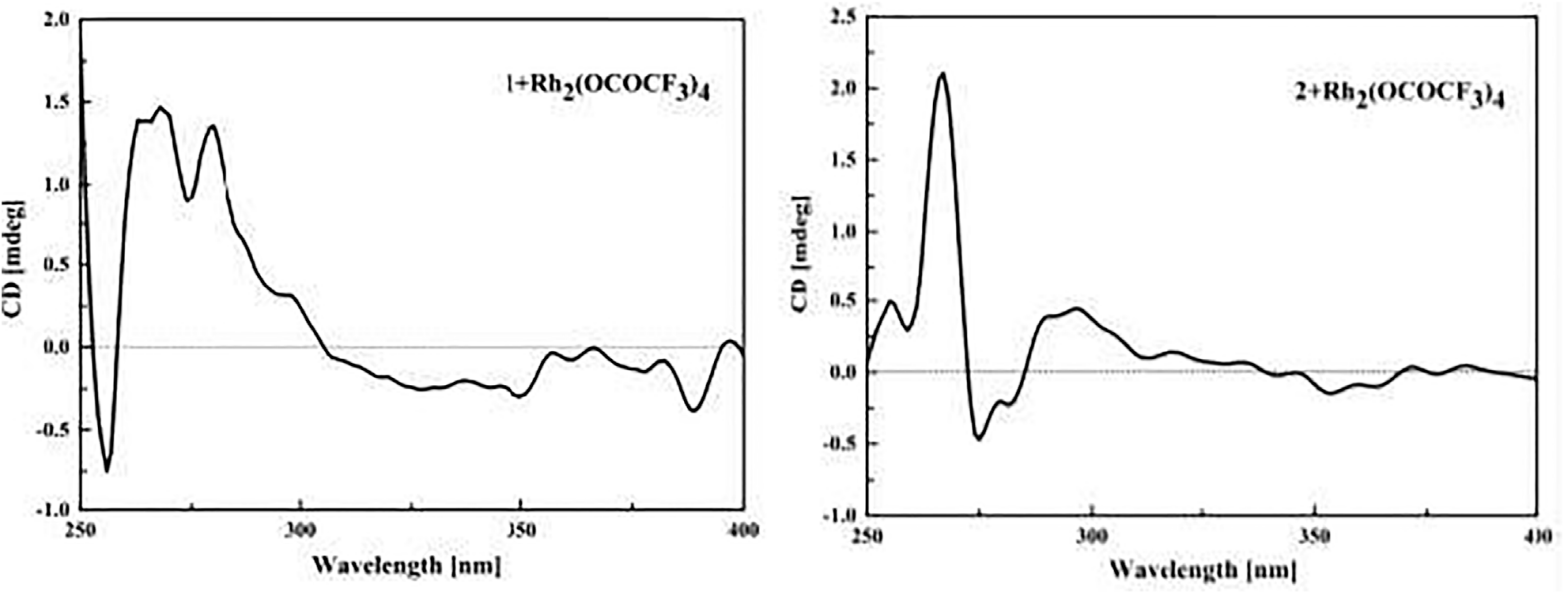
ECD spectra of the Rh complex of **1** and **2** with the inherent ECD spectra subtracted.

Compound **1** was isolated as a yellow amorphous powder with the molecular formula of C_14_H_18_O_6_ established by HRESIMS [*m/z* 305.1006 (M + Na)^+^]. The analysis of its ^1^H, ^13^C, and HSQC NMR spectra (see Supplementary Figures S1–S3 in the Supplementary Material) revealed the presence of six aromatic carbons (three of which were protonated), four methylene groups, one methyl group (*δ*_C/H_ 24.0/1.30), one oxygenated quaternary carbon (*δ*_C_ 77.7), and two carboxylic carbons (*δ*_C_ 177.4 and 170.1). These data combined with the molecular formula suggested four unobserved exchangeable protons, which plus the above data accounted for all the ^1^H and ^13^C NMR resonances for **1**. The hydroxyl groups were located at C-1, C-9, C-12, and C-14 by default supported by the chemical shift values for C-1 (*δ*_C_ 159.4), C-9 (*δ*_C_ 77.7), C-12 (*δ*_C_ 177.4), and C-14 (*δ*_C_ 170.1). The ^1^H–^1^H coupling patterns for the three aromatic protons, H-3 at *δ*_H_ 7.80 (1H, d, *J* = 2.1 Hz), H-5 at *δ*_H_ 7.75 (1H, dd, *J* = 8.5, 2.1 Hz), and H-6 at *δ*_H_ 6.79 (1H, d, *J* = 8.5 Hz), suggested the presence of a 1,2,4-trisubstituted benzene ring, which was confirmed by relevant ^1^H–^1^H COSY and HMBC correlations (Figure 2). The HMBC correlation from H-3 and H-5 to C-14 suggested a carboxylic carbon C-14 attached to C-4 (*δ*_C_123.2) directly. The COSY correlations (in Figure 2) from H_2_-7 (*δ*_H_ 2.84) to H_2_-8 (*δ*_H_ 1.85) and from H_2_-10 (*δ*_H_ 2.01, 1.93) to H_2_-11 (*δ*_H_ 2.46), along with the key HMBC correlations (in Figure 2) from H_2_-10 to C-8 (*δ*_C_ 31.7), from H_2_-10 and H_2_-11 to C-12, and from H_3_-13 (*δ*_H_ 1.30) to C-8, C-9, and C-10 (*δ*_C_ 36.0), indicated a 4-hydroxy-4-methylhexanoic acid fragment. The above two fragments were connected by the key HMBC correlations from H_2_-8 to C-2 (*δ*_C_ 122.2) and from H_2_-7 to C-1 and C-3 (*δ*_C_ 132.9). Thus, the planar structure of **1** was determined as shown in Figure 1, and it was named as cladoslide A. An Rh_2_(OCOCF_3_)_4_-induced electronic circular dichrosim (ECD) experiment (Frelek and Szczepek, 1999) was conducted in order to determine the absolute configuration of the C-9 chiral tertiary alcohol. The induced negative Cotton effect at approximately 350 nm (Figure 3) suggested the 9*S* configuration of **1** based on the bulkiness rule (Gerards and Snatzke, 1990; Frelek and Szczepek, 1999).

Compound **2** was isolated as a yellow oil, whose molecular formula was determined as C_15_H_20_O_6_ by HRESIMS [*m/z* 319.1139 (M + Na)^+^]. A detailed comparison of 1D NMR data of **2** (Table 1) with those of **1** indicated that **2** has a very similar chemical structure to that of **1**. The only difference between them was that the carboxyl group attached to C-11 in **1** was replaced by a carbomethoxy group in **2**, as evidenced by the presence of a methoxyl group (*δ*_C/H_ 52.2/3.65) in ^1^H and ^13^C NMR spectra of **2** and their difference of the molecular formula, along with the key HMBC correlations (in Figure 2) from H-10 (*δ*_H_ 2.03/1.94), H-11(*δ*_H_ 2.50), and H_3_-15 (*δ*_H_ 3.65) to C-12 (*δ*_C_ 175.8). Therefore, the planar chemical structure of **2** was elucidated as shown in Figure 1 and named as cladoslide B. The 9*S* configuration of **2** was also determined according to an induced negative Cotton effect at approximately 350 nm (Figure 3) by an Rh_2_(OCOCF_3_)_4_-induced ECD experiment (Gerards and Snatzke, 1990; Frelek and Szczepek, 1999).

Compound **3** was obtained as a yellow amorphous powder, and it displayed a positive response toward *Dragendorff*’s reagent. Its molecular formula was assigned as C_19_H_19_N_3_O_5_ by HRESIMS [*m/z* 392.1227 (M + Na)^+^]. The UV spectrum of **3** displayed the characteristic absorption maxima of β-carboline chromophore at 362, 278, and 217 nm (Chen et al., 2010; Cao et al., 2012). Interpretation of the ^1^H NMR, ^13^C NMR, and HSQC spectroscopic data of **3** (Table 2) displayed resonances for two exchangeable protons (*δ*_H_ 12.2 and 9.59), eleven aromatic carbons (five of which were protonated), two methyl groups (*δ*_C/H_ 23.5/0.98 and *δ*_C/H_ 21.8/0.96), one methylene group (*δ*_C/H_ 40.69/1.87, 1.77), two sp^3^ methine groups (one of which was heteroatom-bonded at *δ*_H/C_ 4.69/50.9), and three carboxyl or amide carbonyls (*δ*_C_ 174.6, 166.2, and 165.2). These data and the two unobserved exchangeable protons accounted for all the ^1^H and ^13^C NMR resonances for **3**. The ^1^H NMR spectra of **3** exhibited vicinally coupled aromatic proton signals at *δ*_H_ 8.47 (1H, d, *J* = 7.8 Hz, H-5), 7.85 (1H, d, *J* = 8.1 Hz, H-8), 7.64 (1H, t, *J* = 7.8 Hz, H-7), and 7.35 (1H, t, *J* = 7.8 Hz, H-6), which combined with the sequential COSY correlations of H-5/H-6/H-7/H-8 were indicative of a 1,2-disubstituted benzene ring of β-carboline alkaloid (Chen et al., 2010; Cao et al., 2012). A downfield aromatic proton singlet at *δ*_H_ 9.14 (s) was assigned as the characteristic H-4 proton signal of the β-carboline alkaloid, evidenced by its ROESY correlation with H-5. The above signals, together with HMBC correlations from H-4 to C-10 (*δ*_C_ 136.5) and C-12 (*δ*_C_ 120.7), from H-5 to C-11 (*δ*_C_ 132.2) and C-12, from H-6 and H-8 to C-12, from H-5 and H-7 to C-13 (*δ*_C_ 142.7), and from 9-NH (*δ*_H_ 12.22) to C-1 (*δ*_C_ 142.3), C-10, C-11, and C-12, suggested the presence of a β-carboline skeleton (Chen et al., 2010; Cao et al., 2012). A comparison of the ^1^H and ^13^C NMR data for **3** (Table 2) with those of the previously reported dichotomine H (Cao et al., 2012) suggested that **3** has a very similar chemical structure to that of dichotomine H (Cao et al., 2012). The main structural difference between them was that the glutamic acid unit in dichotomine H was replaced by leucine in **3**, as evidenced by the sequential COSY correlations of H-15 (NH, *δ*_H_ 9.59)/H-16 (*δ*_H_ 4.69)/H_2_-18 (*δ*_H_ 1.87, 1.77)/H-19 (*δ*_H_ 1.76), H-20 (*δ*_H_ 0.98)/H-19, and H-21 (*δ*_H_ 0.96)/H-19, together with HMBC correlations from H_2_-18 and H-16 to C-17 (*δ*_C_ 174.6). Moreover, the leucine unit was connected to C-14 (*δ*_C_ 165.2) rather than C-22 (*δ*_C_ 166.2) on the basis of the obvious ROESY correlations between 9-NH (*δ*_H_ 12.22) and H-16 (*δ*_H_ 4.69). A carboxyl group (C-22) was attached to C-3 (*δ*_C_ 131.0) in **3** according to the key HMBC correlation from H-4 to C-3 and C-22 combined with the molecular formula. The absolute configuration of the leucine was identified as L-leucine by Marfey’s method (Marfey, 1984). The mixture obtained after hydrolyzing compound **3** and further derivatization with L-FDAA was analyzed by HPLC-DAD. The derivatives of two authentic leucine samples (L- and D-) were also prepared and analyzed by HPLC-DAD (see Supplementary Figure S26 in Supplementary Material). The chromatogram of the derivative of **3** displayed the peak with the retention time (t_*R*_ 24.28 min), which was consistent with the retention time and the UV spectra obtained for the derivative of L-Leu (t_*R*_ 24.28 min) and different from the retention time obtained for the derivative of D-Leu (t_*R*_ 28.59 min). Finally, the leucine moiety in **3** was unambiguously identified as L-Leu, and the structure of compound **3** was elucidated as shown in Figure 1, which was named cladospomine.

Compound **4** was obtained as a yellow oil and possessed a molecular formula C_10_H_13_NO_3_ based on a prominent sodium adduct ion peak at *m/z* 218.0779 [M + Na]^+^ in the HRESIMS spectrum. Its ^1^H, DEPTQ, and HSQC NMR spectra (see Supplementary Figures S20–S22 in Supplementary Material) showed signals for three sp^2^ methine groups (*δ*_C/H_ 109.8/6.31, *δ*_C/H_ 137.4/7.53, and *δ*_C/H_ 117.8/6.40), three methylene groups (*δ*_C/H_ 48.4/4.03, *δ*_C/H_ 24.2/2.03, and *δ*_C/H_ 30.2/2.36), one methyl group (*δ*_C/H_ 19.8/2.25), one amide carbonyl signal (*δ*_C_ 163.3), one carboxyl carbonyl signal (*δ*_C_ 174.9), and one sp^2^ quaternary carbon signal (*δ*_C_ 153.2). The sequential COSY correlations from H_2_-7 (*δ*_H_ 4.03) through H_2_-9 (*δ*_H_ 2.36) combined with the HMBC correlation from H_2_-8 and H_2_-9 to C-10 (*δ*_C_ 174.9) indicated a butyric acid fragment. The COSY correlations from H-5 (*δ*_H_ 6.31) to H-6 (*δ*_H_ 7.53), together with the HMBC correlations from H_3_-11 (*δ*_H_ 2.25) to C-3 (*δ*_C_ 117.8), C-4 (*δ*_C_ 153.2), and C-5 (*δ*_C_ 109.8), from H-3 (*δ*_H_ 6.40) and H-6 to C-2 (*δ*_C_ 163.3), and from H-5 to C-3 displayed a 4-methylpyridin-2(1*H*)-one fragment. Finally, the key HMBC correlations from H_2_-7 to C-2 and C-6 (*δ*_C_ 137.4) connected above two fragments. Thus, the chemical structure of compound **4** was identified as shown in Figure 1 and named as cladoslide C.

The chemical structures of the previously reported *N*-acetyl-β-oxotryptamine (**5**) (Martínez-Luis et al., 2012), (4*S*,5*S*,11*R*)-*iso*-cladospolide B (**6**) (Reddy et al., 2012), (4*S*,5*S*,11*S*)-*iso*-cladospolide B (**7**) (Reddy et al., 2012), and (4*R*,5*S*,11*R*)-*iso*-cladospolide B (**8**) (Franck, et al., 2001) were identified by comparison of their spectroscopic data (see Supplementary Tables S1, S2 in Supplementary Material) with those in the literature.

**FIGURE 4:**
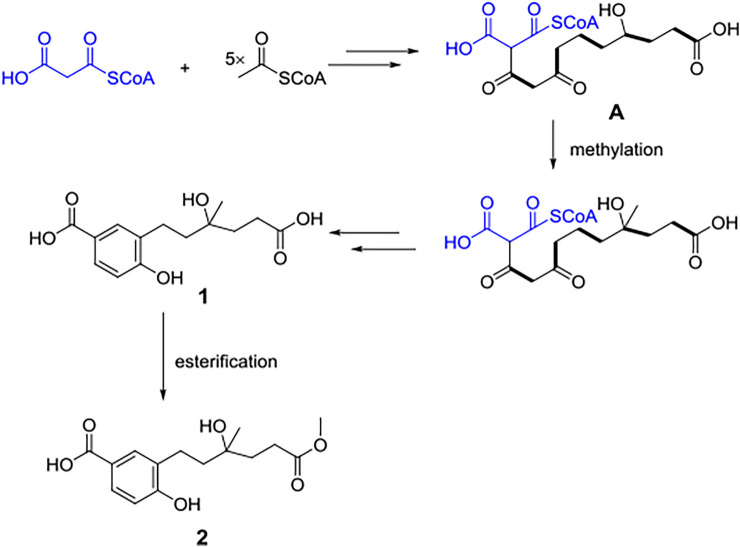
Hypothetical biogenetic pathway of compounds **1** and **2**.

Compounds **1** and **2** were postulated to be produced biogenetically from the polyketide pathway. Condensation and redox reaction between one malony CoA unit and five acetyl-CoA units formed intermediate (**A**), which further underwent methylation to form compound **1**. Then, **1** underwent esterification to afford **2** (Figure 4).

### Biological Activity

Compounds **1**–**3** and **5**–**8** were tested for their cytotoxicity against Hela, BEL-7402, K562, and SGC-7901 cell lines and α-glycosidase inhibitory activity (Table 3). Compound **1** showed cytotoxicity against the K562 cell line with an IC_50_ value of 13.10 ± 0.08 μM. Besides, compounds **1** and **5** exhibited inhibitory activity against α-glycosidase with IC_50_ values of 0.32 ± 0.01 mM and 0.17 ± 0.01 mM, respectively.

**TABLE 3:** IC_50_ values of cytotoxicity and α-glycosidase inhibitory activity of compounds **1**–**3** and **5**–**8**.

Compounds	IC_50_ (μM)				IC_50_ (mM)
	Hela	BEL-7042	K562	SGC-7901	α-Glycosidase
**1**	>100	>100	13.10 ± 0.08	>100	0.32 ± 0.01
**2**	>100	>100	>100	>100	>1.0
**3**	>100	>100	>100	>100	>1.0
**5**	>100	>100	>100	>100	0.17 ± 0.01
**6**	>100	>100	>100	>100	>1.0
**7**	>100	>100	>100	>100	>1.0
**8**	>100	>100	>100	>100	>1.0
Adriamycin	0.28 ± 0.01	0.47 ± 0.01	0.10 ± 0.01	0.22 ± 0.01	ND^a^
Acarbose	ND^a^	ND^a^	ND^a^	ND^a^	0.72 ± 0.01

a Not detected.

## Conclusion

In conclusion, four new compounds (**1**–**4**) were isolated from the rice medium culture of the mangrove-derived fungus *Cladosporium* sp. HNWSW-1, along with four previously reported *N*-acetyl-β-oxotryptamine (**5**), (4*S*,5*S*,11*R*)-*iso*-cladospolide B (**6**), (4*S*,5*S*,11*S*)-*iso*-cladospolide B (7), and (4*R*,5*S*,11*R*)-*iso*-cladospolide B (**8**). Compound **1** showed cytotoxicity against the K562 cell line with an IC_50_ value of 13.10 ± 0.08 μM. Moreover, compounds **1** and **5** exhibited inhibitory activity against α-glycosidase with IC_50_ values of 0.32 ± 0.01 mM and 0.17 ± 0.01 mM, respectively.

## Data Availability

The datasets presented in this study can be found in online repositories. The names of the repository/repositories and accession number(s) can be found in the article/Supplementary Material.
