# Supplementary material for: Metabolites From the Mangrove-Derived Fungus Cladosporium sp. HNWSW-1
Source: Front Chem. 2021 Dec 16;9:773703. doi: 10.3389/fchem.2021.773703 (PMC8717711; doi:10.3389/fchem.2021.773703)
Supplement: Supplementary file 1 [file DataSheet1.docx]

**Supplementary Material**

**Table of Contents**

**Supplementary Figure 1.** The ^1^H NMR spectrum of compound **1** in CD_3_OD-*d*_4_ (500

Hz)……………………………………………………………………………………..3

**Supplementary Figure 2.** The ^13^C NMR and DEPT135 spectra of compound **1** in CD_3_OD-*d*_4_ (125MHz)………………………………………………………………….4

**Supplementary Figure 3.** The HSQC spectrum of compound **1** in CD_3_OD-*d*_4_……..5

**Supplementary Figure 4.** The ^1^H-^1^H COSY spectrum of compound **1** in CD_3_OD -*d*_4_……………………………………………………………………………………6

**Supplementary Figure 5.** The HMBC spectrum of compound **1** in CD_3_OD-*d*_4_……..7

**Supplementary Figure 6.** The HRESIMS spectrum of compound **1**………………8

**Supplementary Figure 7.** The ^1^H NMR spectrum of compound **2** in CD_3_OD-*d*_4_ (500 MHz) ……………………………………………………………………………….9

**Supplementary Figure 8.** The ^13^C NMR and DEPT135 spectra of compound **2** in CD_3_OD-*d*_4_ (125MHz)……………………………………………………………….10

**Supplementary Figure 9.** The HSQC spectrum of compound **2** in CD_3_OD-*d*_4_……11

**Supplementary Figure 10.** The ^1^H-^1^H COSY spectrum of compound **2** in CD_3_OD- *d*_4_……………………………………………………………………………………..12

**Supplementary Figure 11.** The HMBC spectrum of compound **2** in CD_3_OD-*d*_4_…13

**Supplementary Figure 12.** The HRESIMS spectrum of compound **2**…………14

**Supplementary Figure 13.** The ^1^H NMR spectrum of compound **3** in DMSO-*d*_6_ (500 MHz)…………………………………………………………………………………15

**Supplementary Figure 14.** The ^13^C NMR and DEPT135 spectra of compound **3** in DMSO-*d*_6_ (125 MHz)…………………………………………………………….16

**Supplementary Figure 15.** The HSQC spectrum of **3** in DMSO-*d*_6_………………...17

**Supplementary Figure 16.** The ^1^H-^1^H COSY spectrum of compound **3** in DMSO-*d*_6_ ……………………………………………………………………………18

**Supplementary Figure 17.** The HMBC spectrum of compound **3** in DMSO-*d*_6_…..19

**Supplementary Figure 18.** The ROESY spectrum of compound **3** in DMSO-*d*_6_….20

**Supplementary Figure 19.** The HRESIMS spectrum of compound **3**……………21

**Supplementary Figure 20.** The ^1^H NMR spectrum of compound **4** in CD_3_OD-*d*_4_ (600 Hz)…………………………………………………………………………….22

**Supplementary Figure 21.** The DEPTQ spectrum of compound **4** in CD_3_OD-*d*_4_ (150Hz)……………………………………………………………………………....23

**Supplementary Figure 22.** The HSQC spectrum of compound **4** in CD_3_OD-*d*_4_…..24

**Supplementary Figure 23.** The ^1^H-^1^H COSY spectrum of compound **4** in CD_3_OD- *d*_4_…………………………………………………………………………….25

**Supplementary Figure 24.** The HMBC spectrum of compound **4** in CD_3_OD-*d*_4_…26

**Supplementary Figure 25**. The HRESIMS spectrum of compound **4**……………...27

**Supplementary Figure 26.** Marfey’s method applying for compound **3**…………...28

**Supplementary Figure 27.** HPLC analysis of compounds **1-3**……………………..29

**Supplementary Table 1.** ^1^H and ^13^C NMR data for **5-8** (500 and 125 MHz, *δ* in ppm)………………………………………………………………………………….30

**Supplementary Table 2 .**Appearance, specific rotation and MS data of **5**-**8**.………..31

**Supplementary Figure 1.** The ^1^H NMR spectrum of compound **1** in CD_3_OD-*d*_4_ (500 MHz)


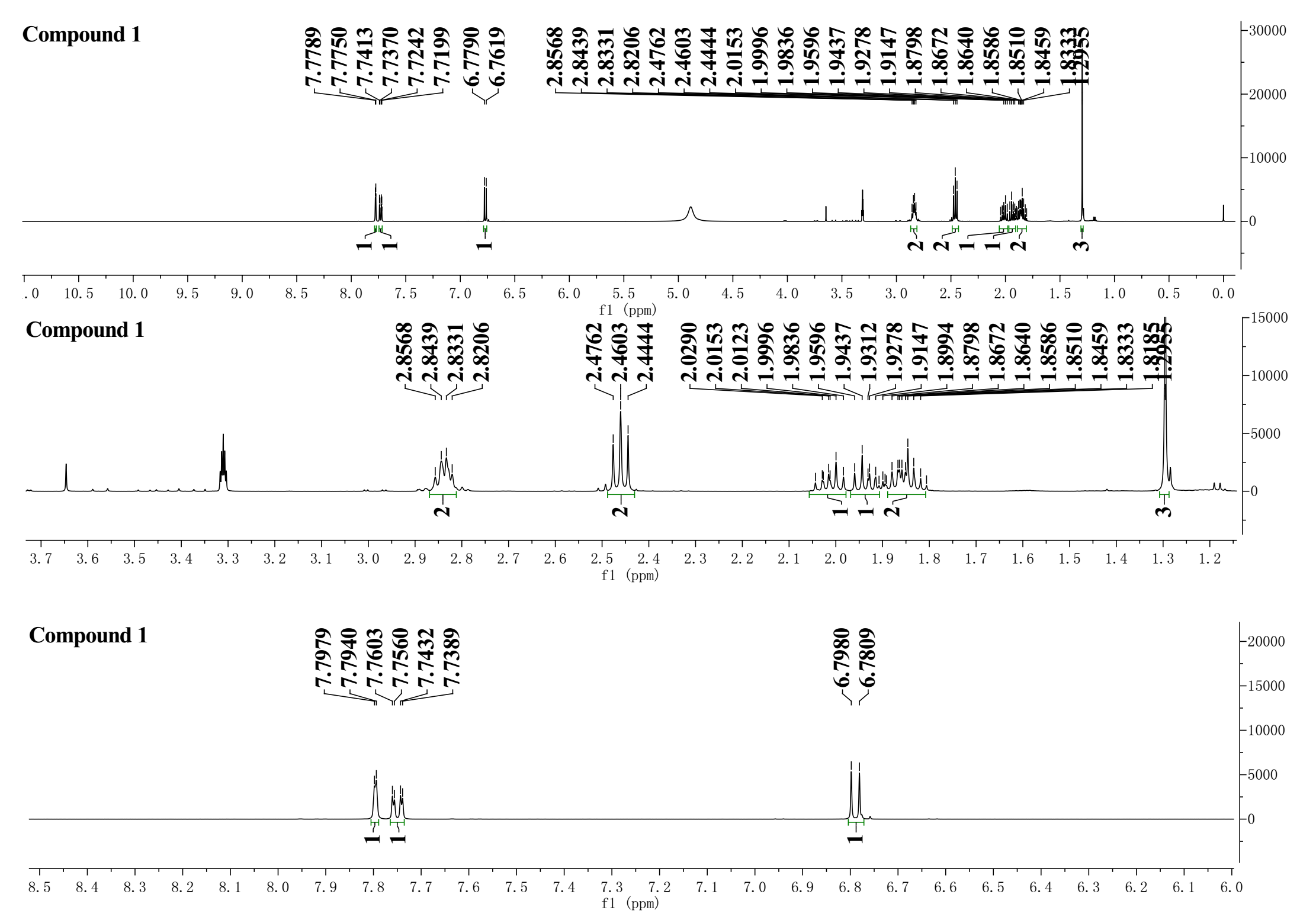


**Supplementary Figure 2.** The ^13^C NMR and DEPT135 spectra of compound **1** in CD_3_OD-*d*_4_ (125MHz)


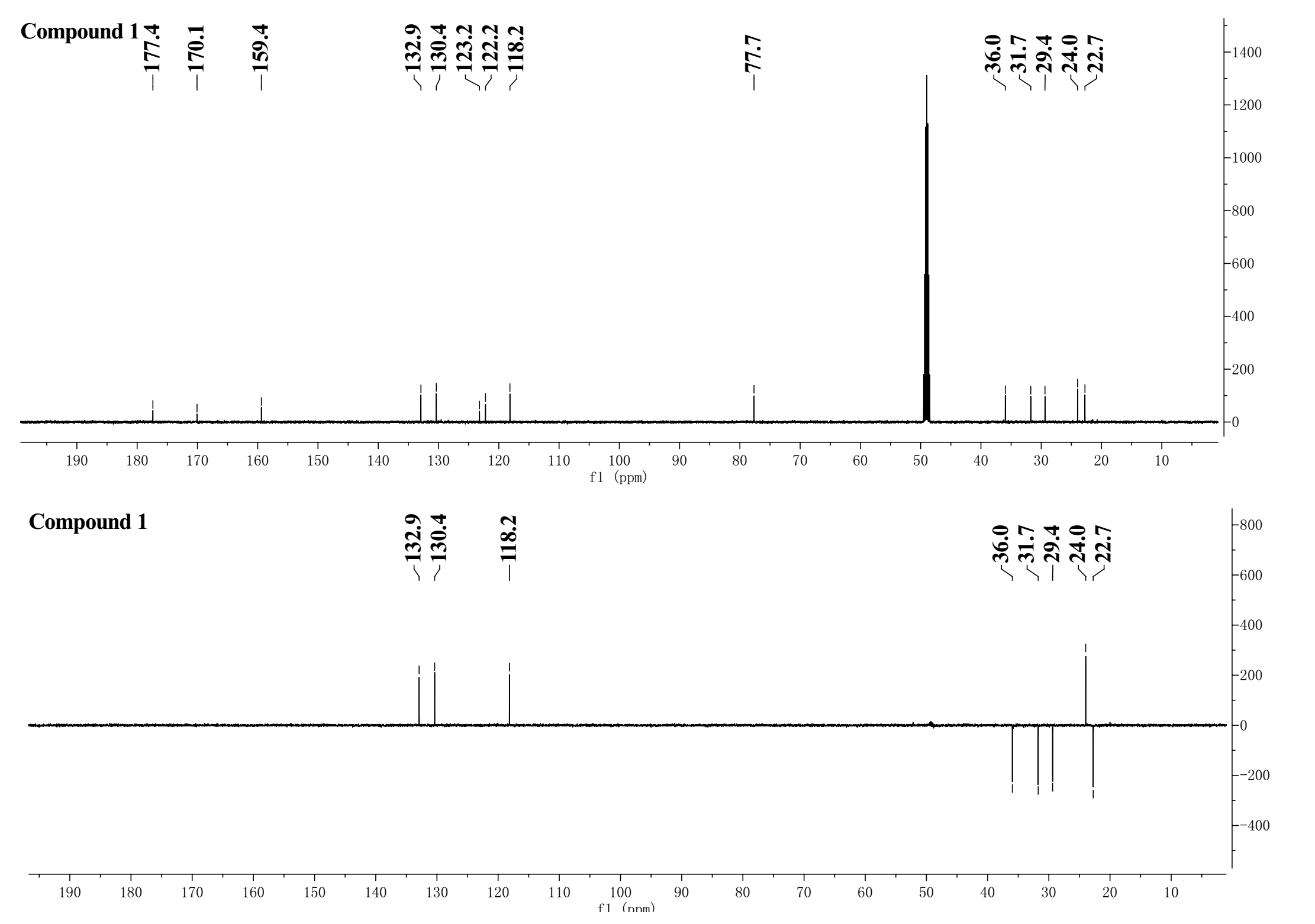


**Supplementary Figure 3.** The HSQC spectrum of compound **1** in CD_3_OD-*d*_4_


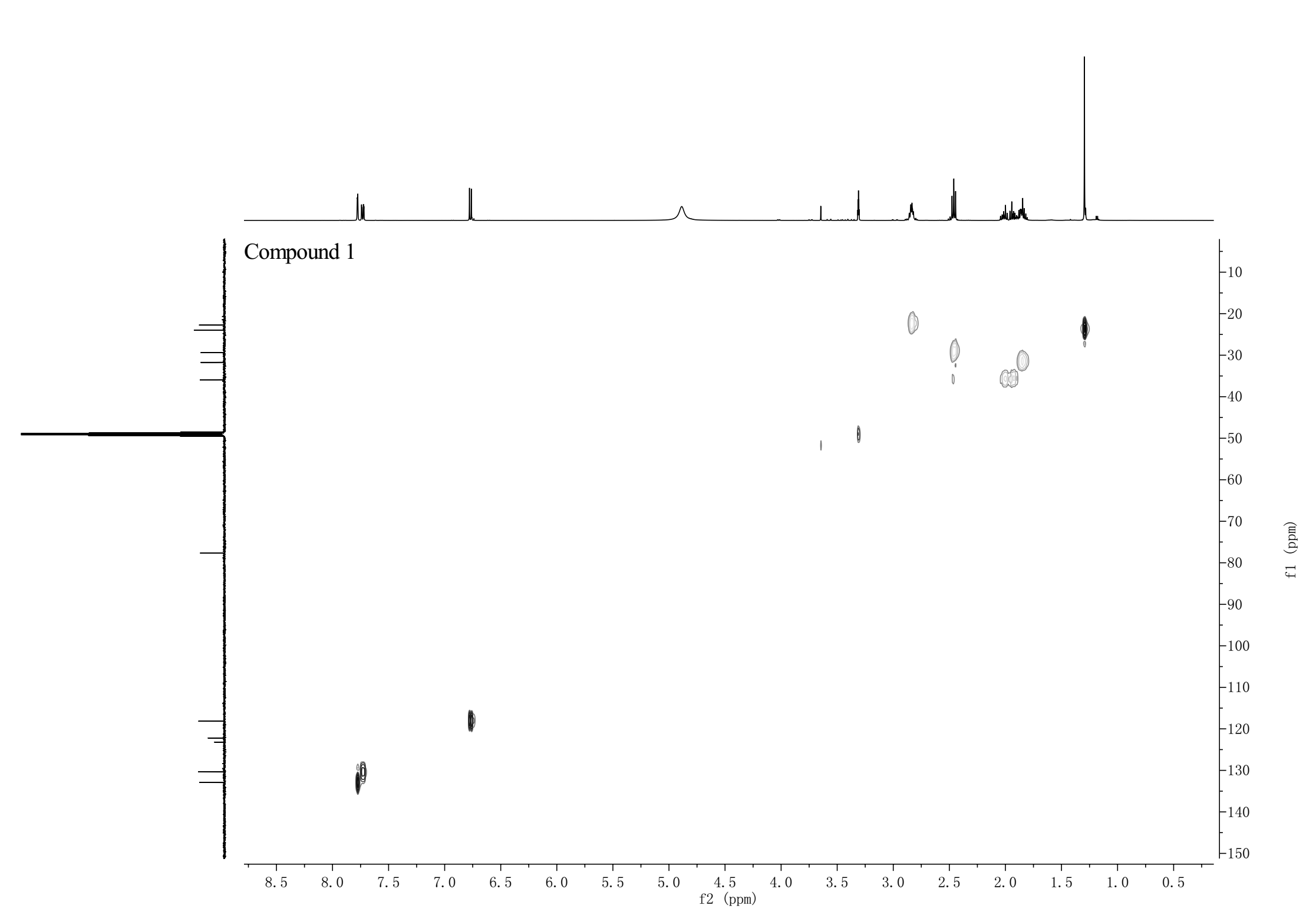


**Supplementary Figure 4.** The ^1^H-^1^H COSY spectrum of compound **1** in CD_3_OD-*d*_4_


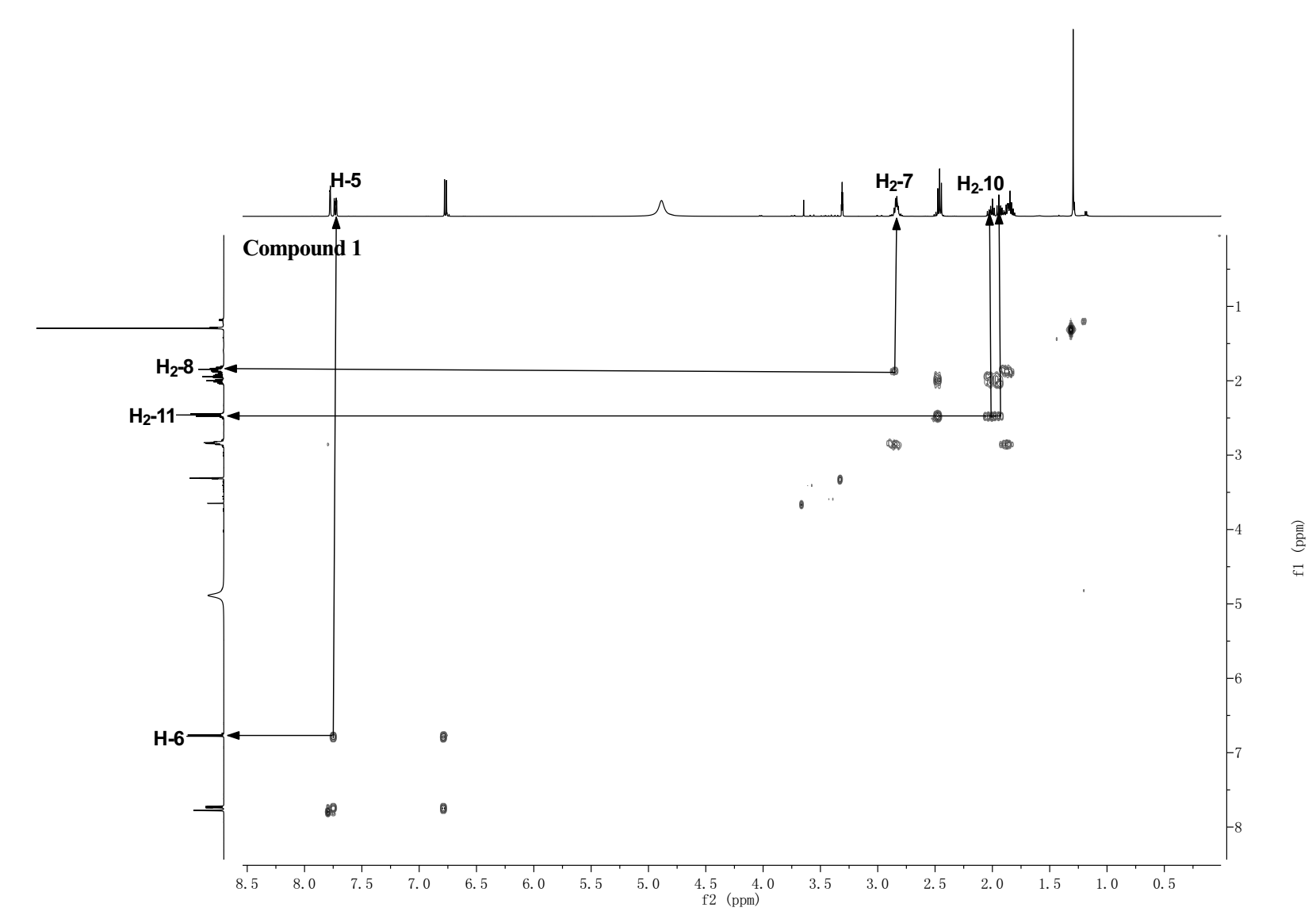


**Supplementary Figure 5.** The HMBC spectrum of compound **1** in CD_3_OD-*d*_4_


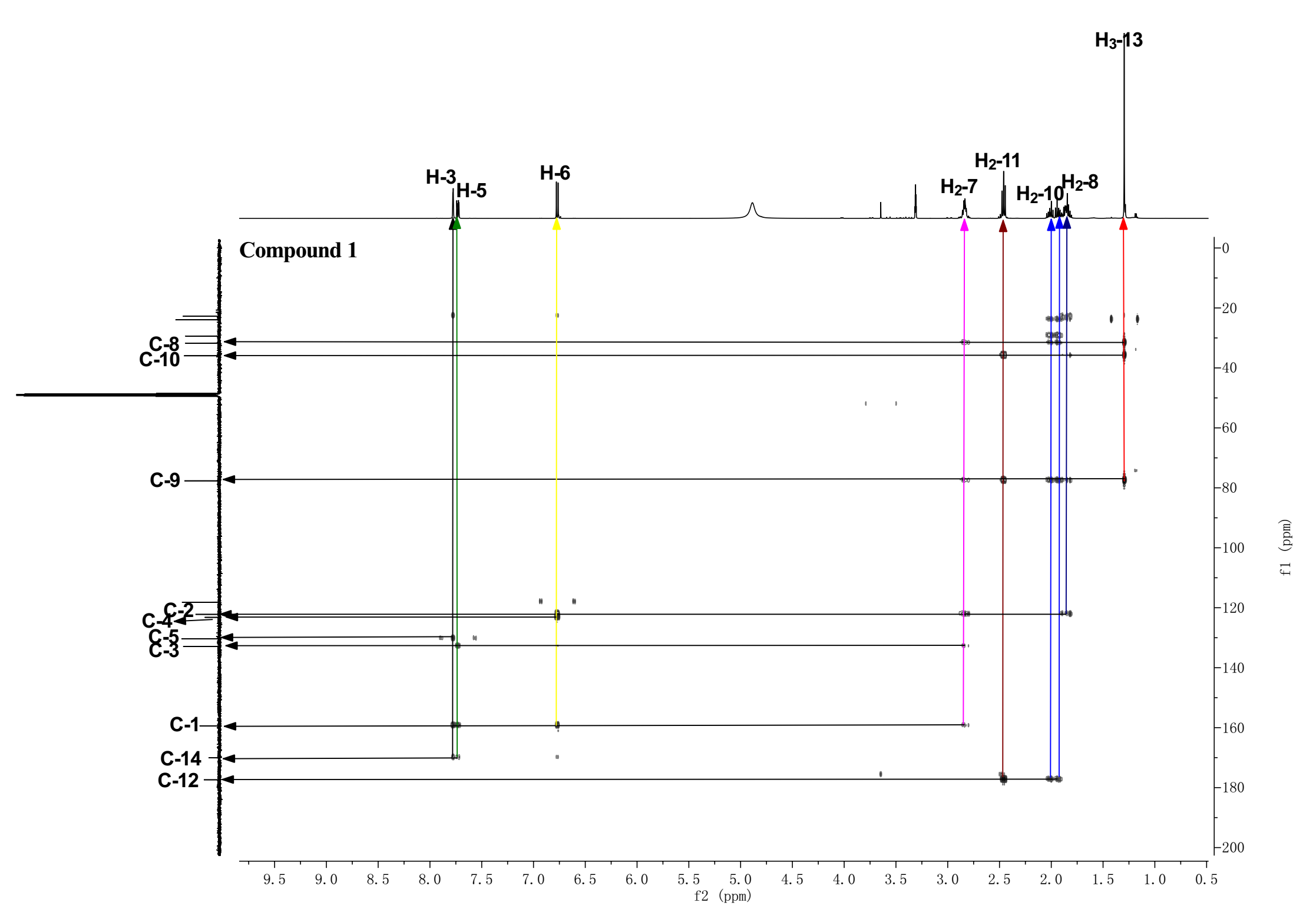

**Supplementary Figure 6.** The HRESIMS spectrum of compound **1**


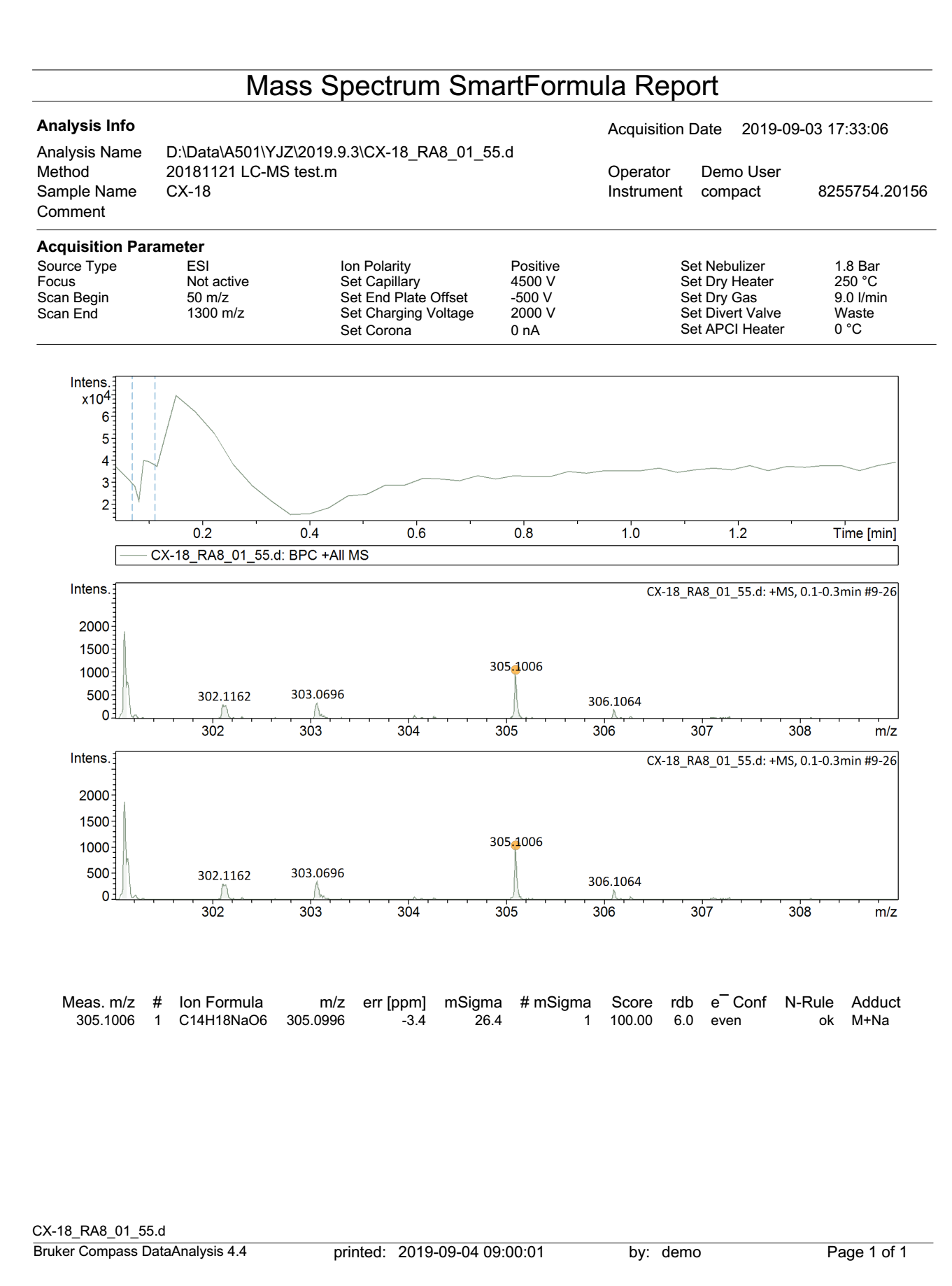


**Supplementary Figure 7.** The ^1^H NMR spectrum of compound **2** in CD_3_OD-*d*_4_ (500 MHz)


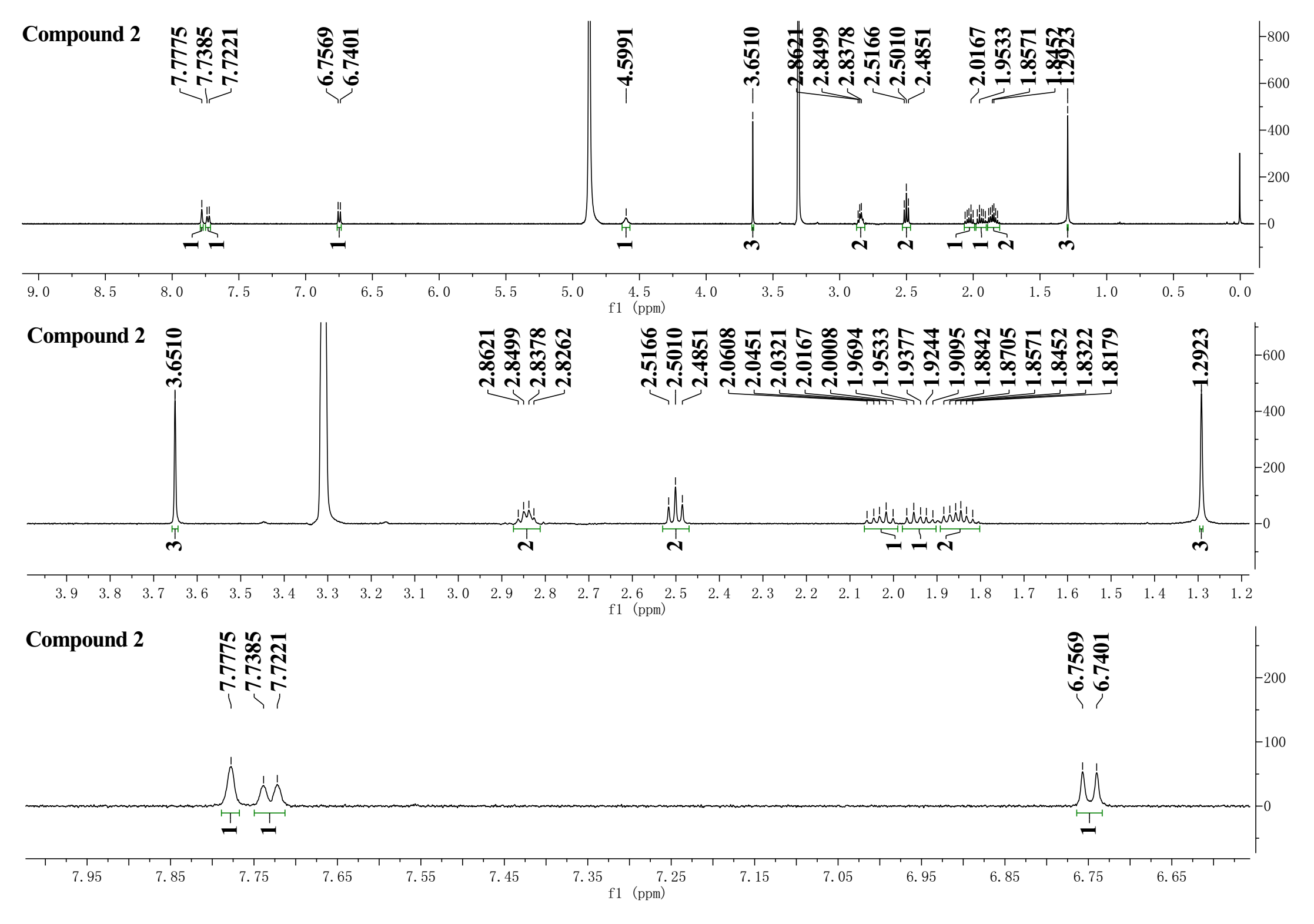


**Supplementary Figure 8.** The ^13^C NMR and DEPT135 spectra of compound **2** in CD_3_OD-*d*_4_ (125 Hz)


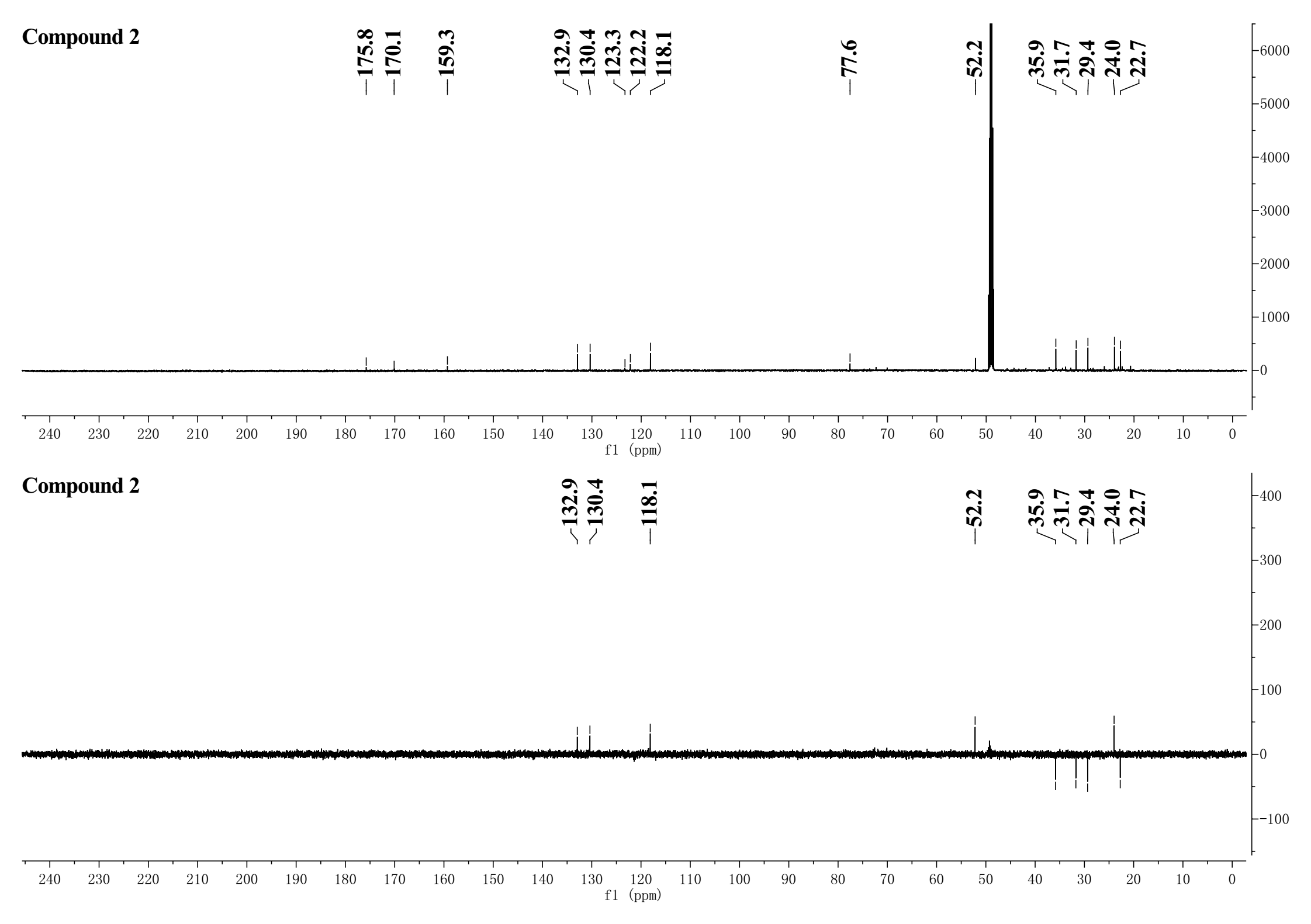


**Supplementary Figure 9.** The HSQC spectrum of compound **2** in CD_3_OD-*d*_4_


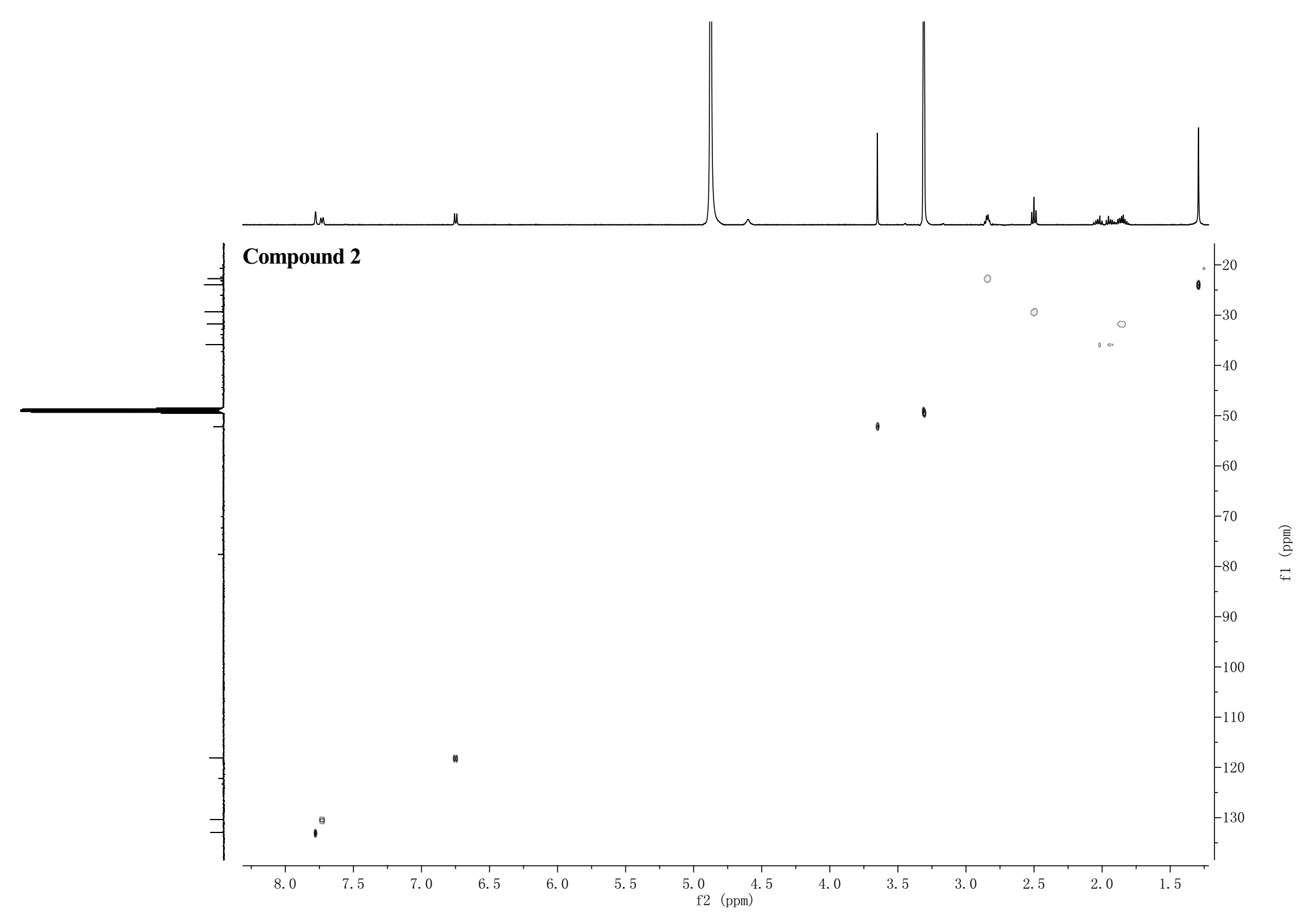


**Supplementary Figure 10.** The ^1^H-^1^H COSY spectrum of compound **2** in CD_3_OD-*d*_4_


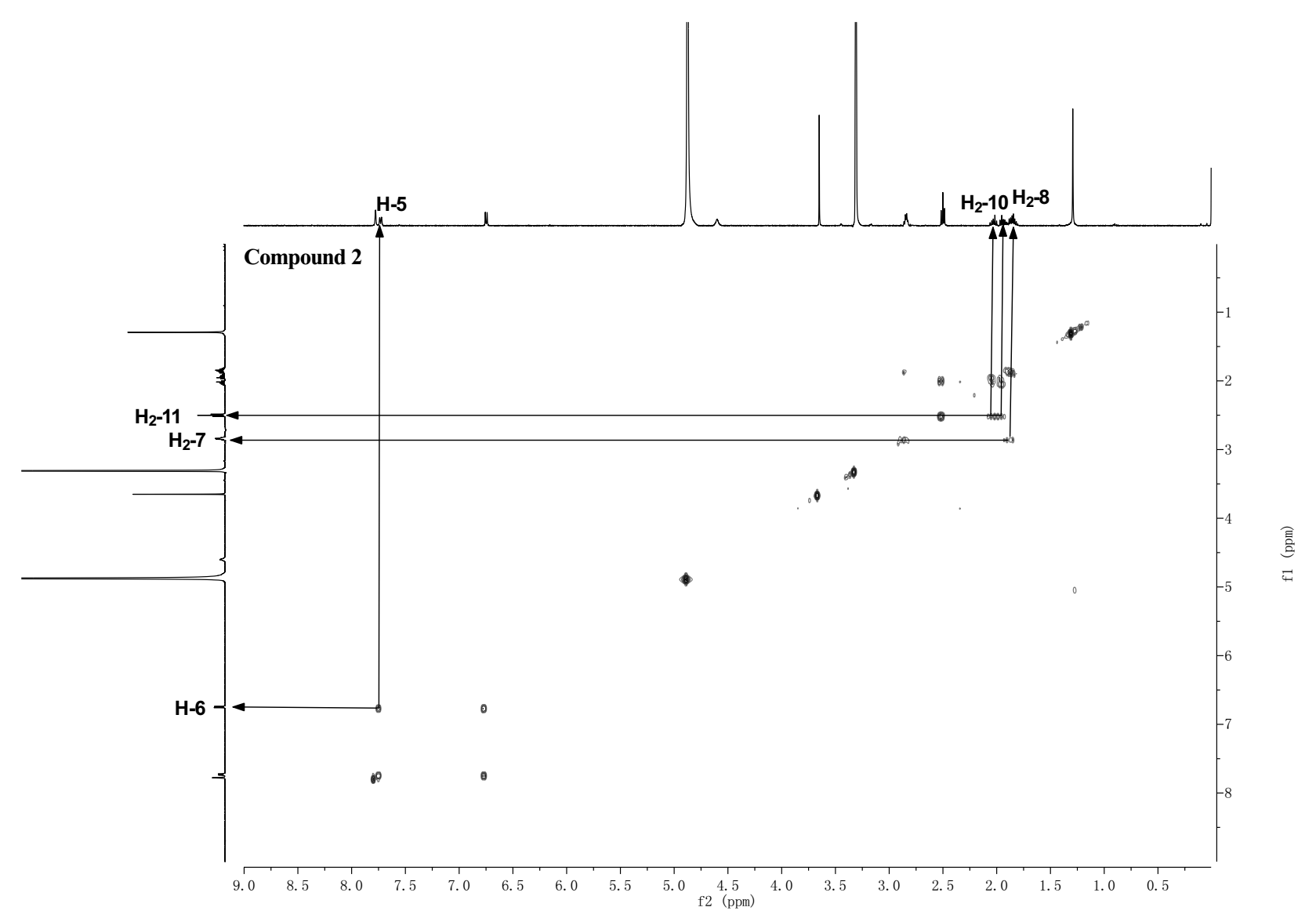


**Supplementary Figure 11.** The HMBC spectrum of compound **2** in CD_3_OD-*d*_4_


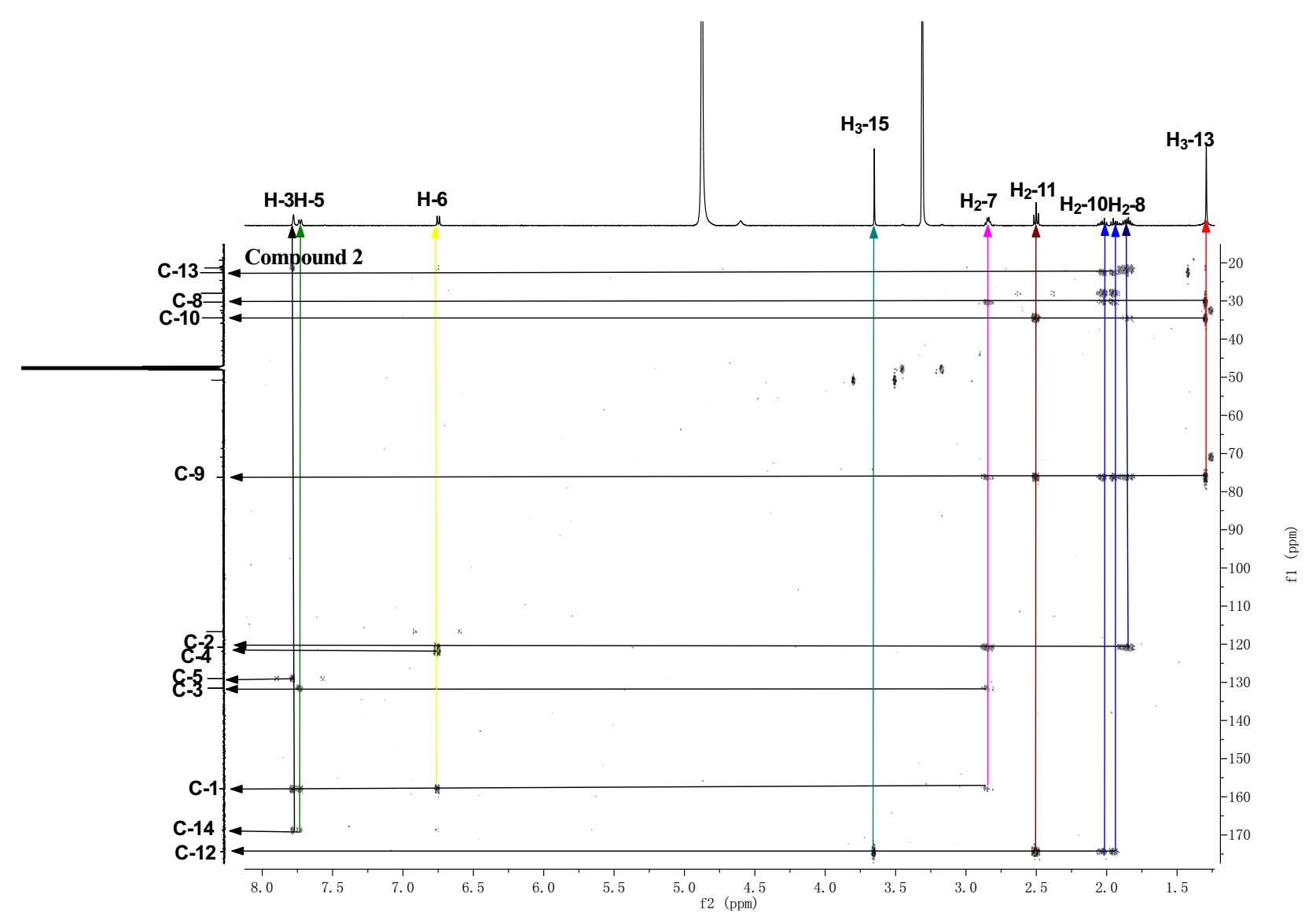

**Supplementary Figure 12.** The HRESIMS spectrum of compound **2**


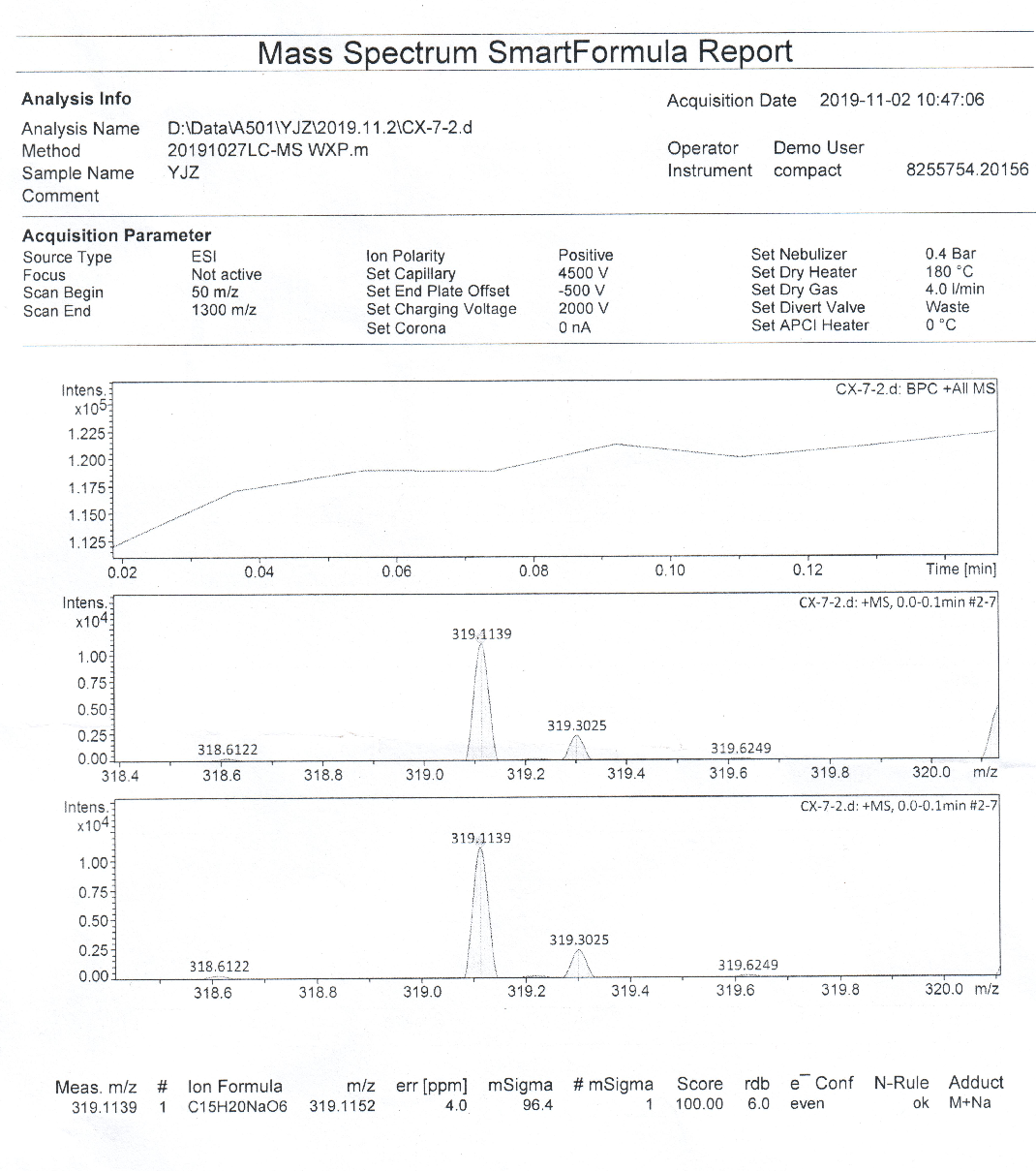


**Supplementary Figure 13.** The ^1^H NMR spectrum of compound **3** in DMSO-*d*_6_ (500 MHz)


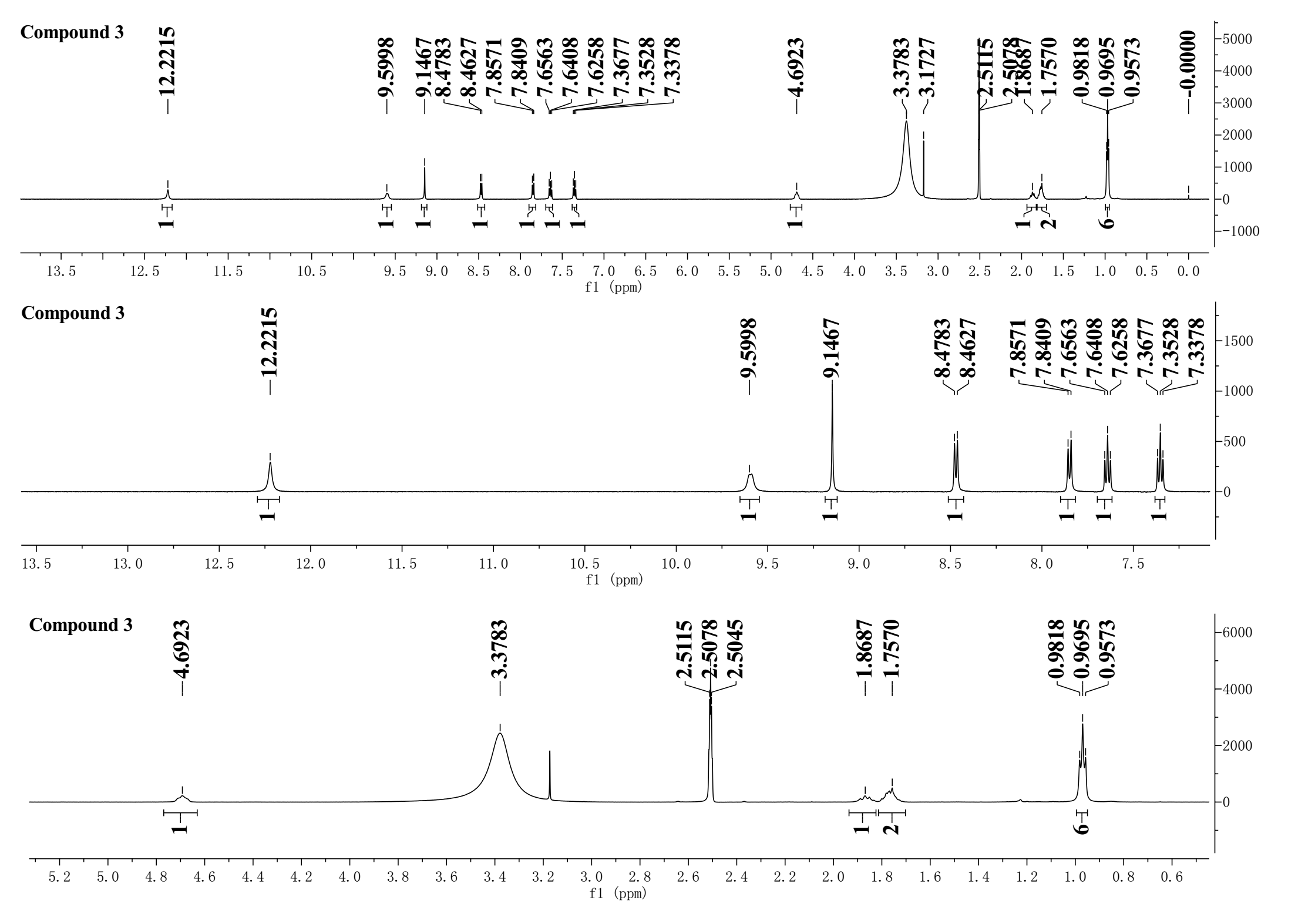


**Supplementary Figure 14.** The ^13^C NMR and DEPT135 spectra of compound **3** in CD_3_OD-*d*_4_ (125 Hz)


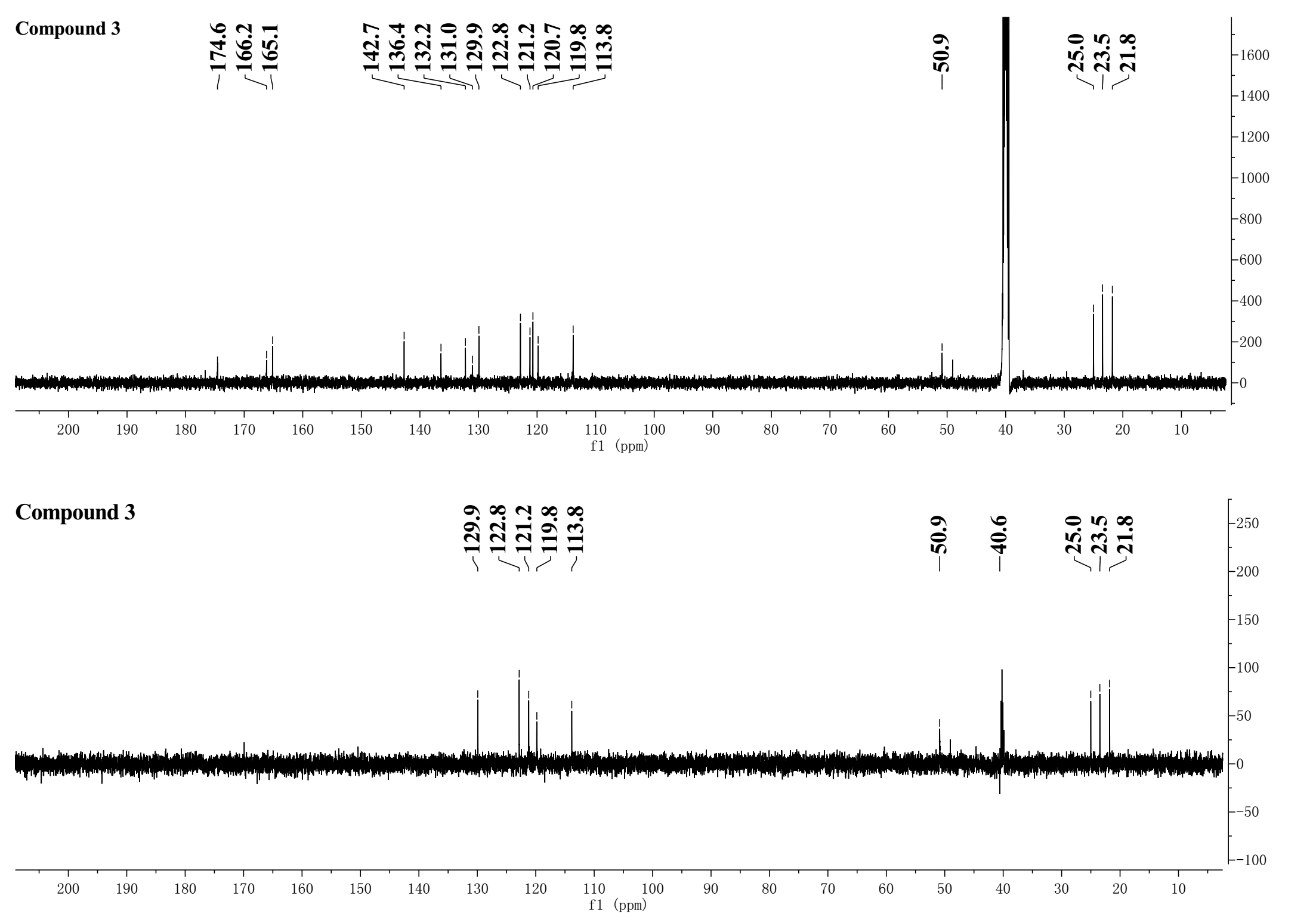


**Supplementary Figure 15.** The HSQC spectrum of compound **3** in DMSO-*d*_6_


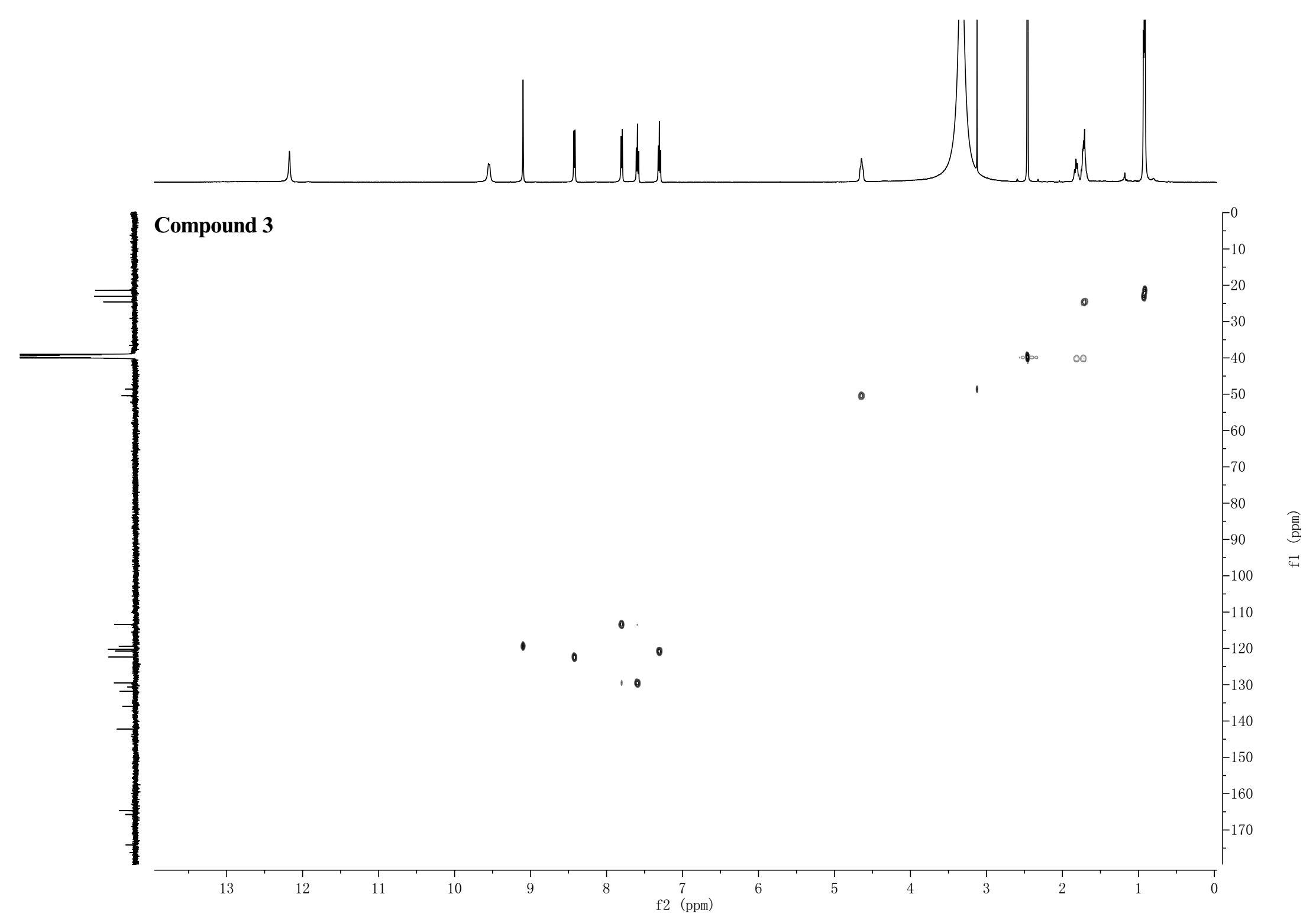


**Supplementary Figure 16.** The ^1^H-^1^H COSY spectrum of compound **3** in DMSO-*d*_6_


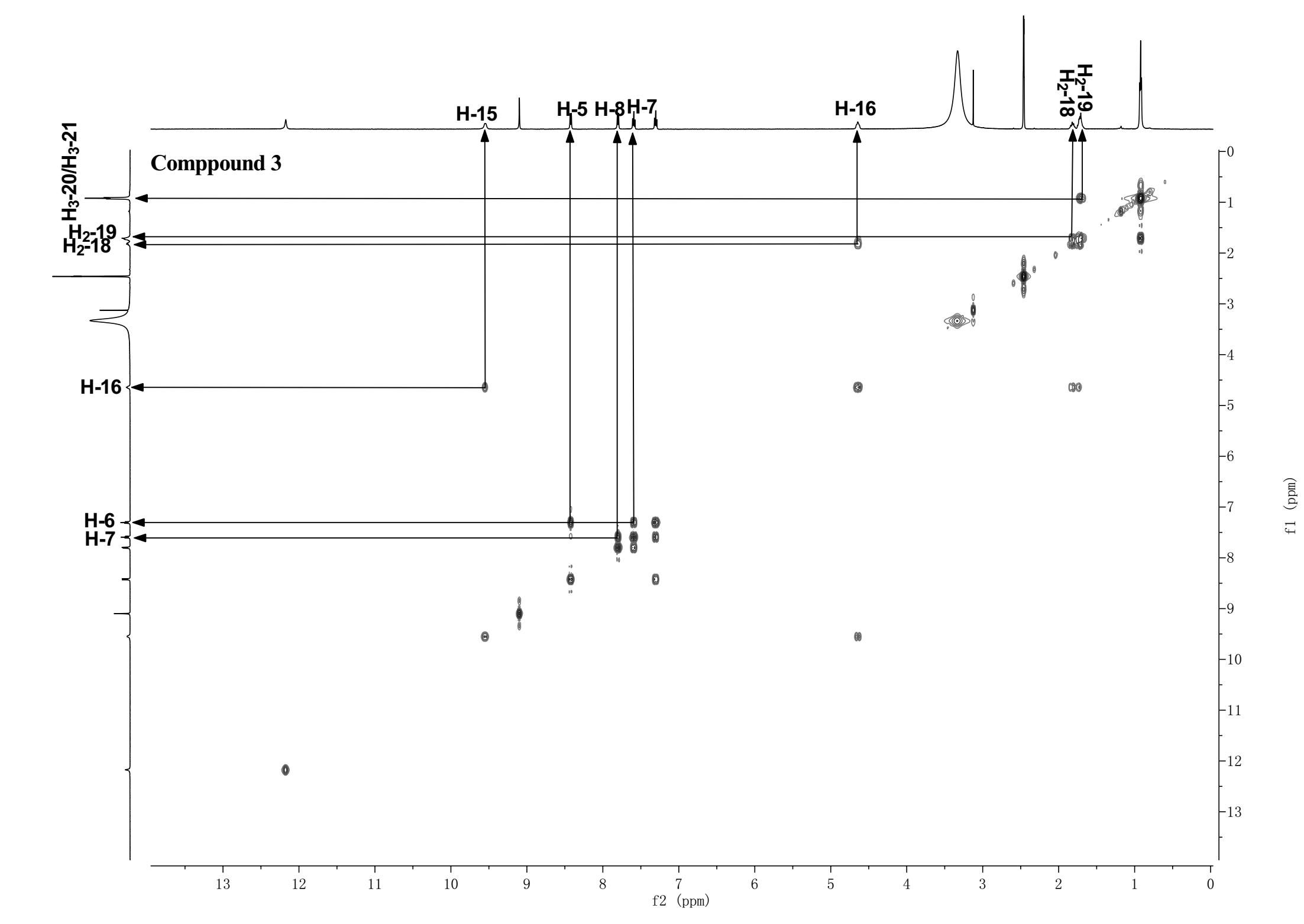


**Supplementary Figure 17**. The HMBC spectrum of compound **3** in DMSO-*d*_6_


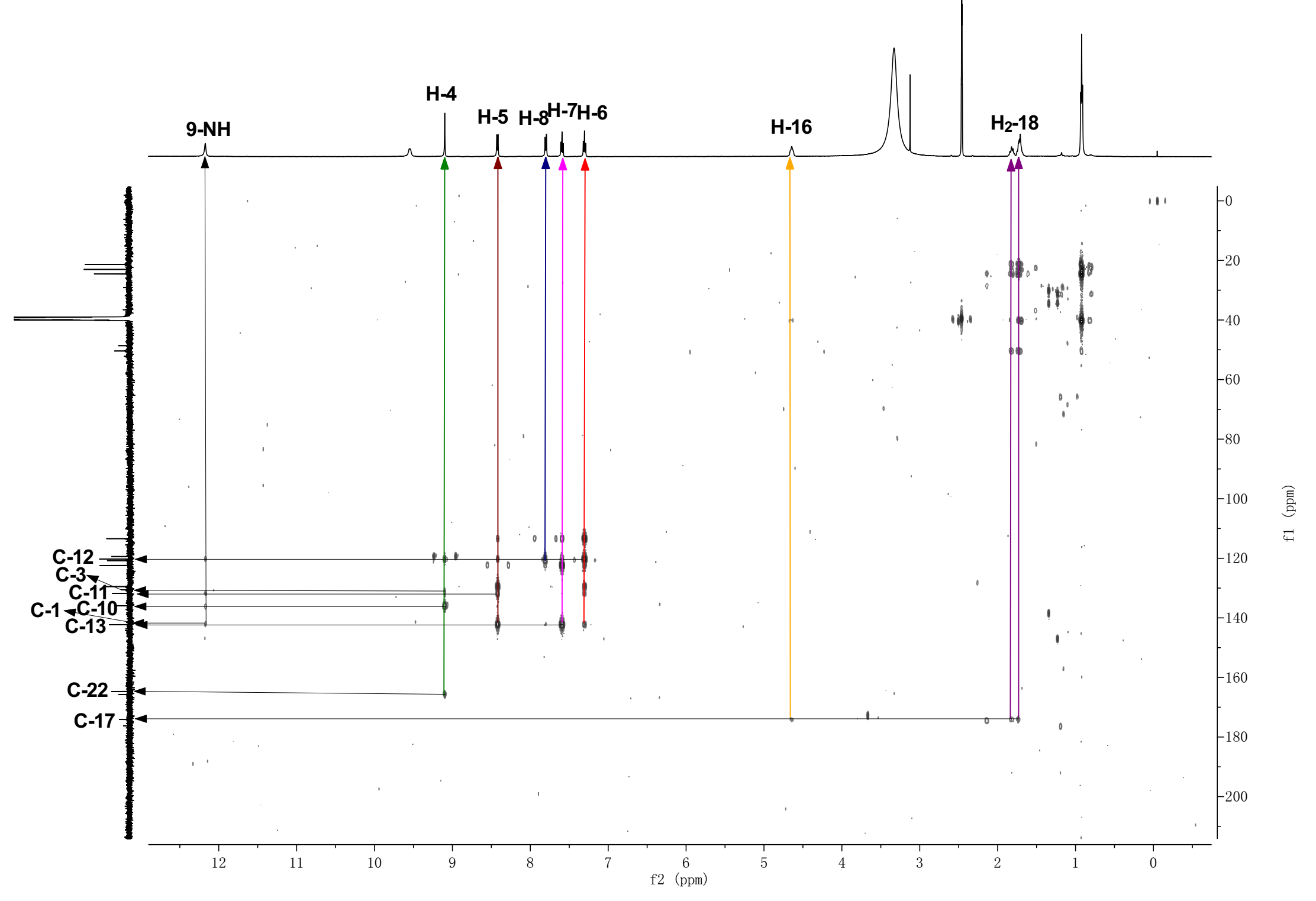

**Supplementary Figure 18.** The ROESY spectrum of compound **3** in DMSO-*d*_6_


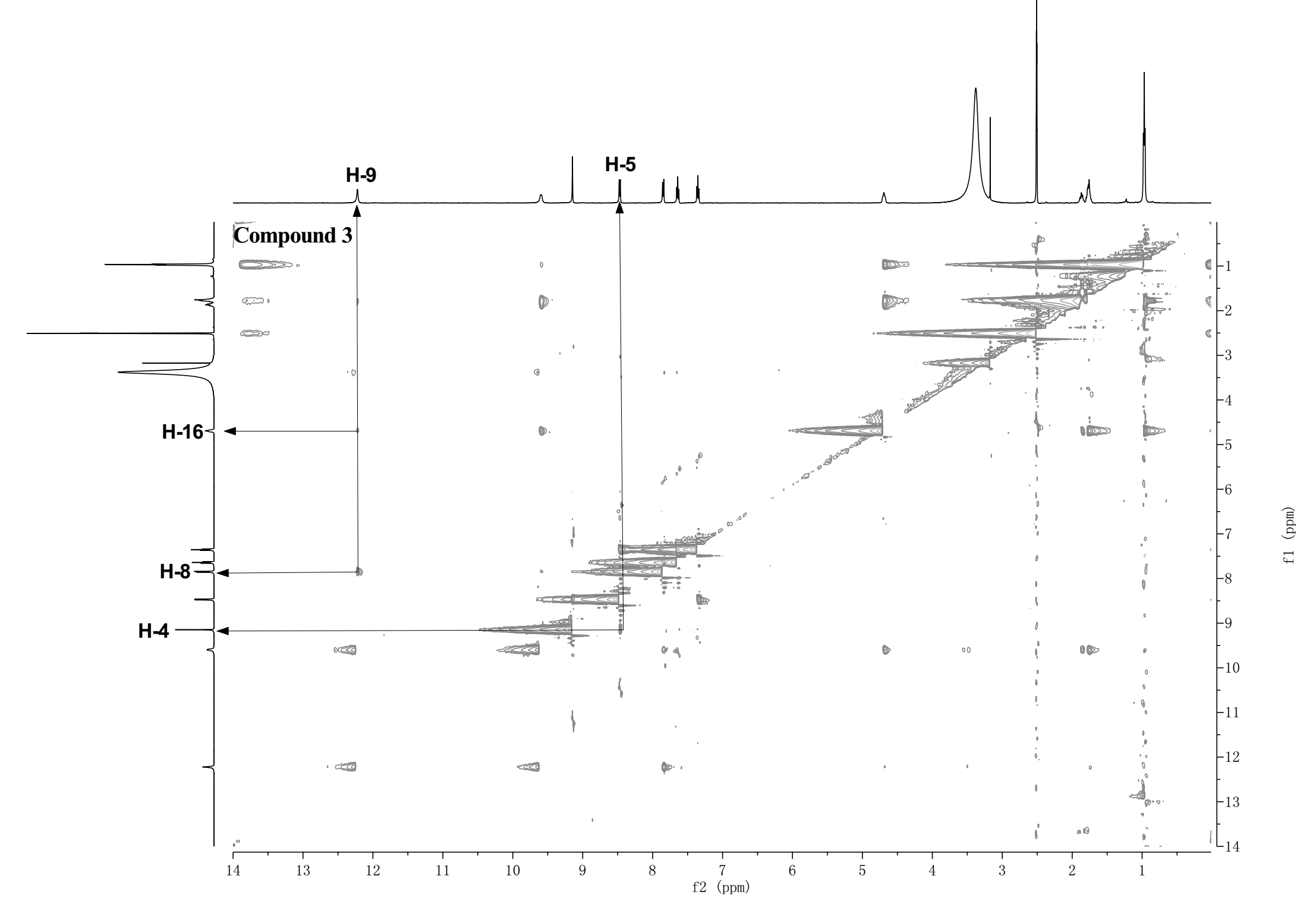

**Supplementary Figure 19.** The HRESIMS spectrum of compound **3**


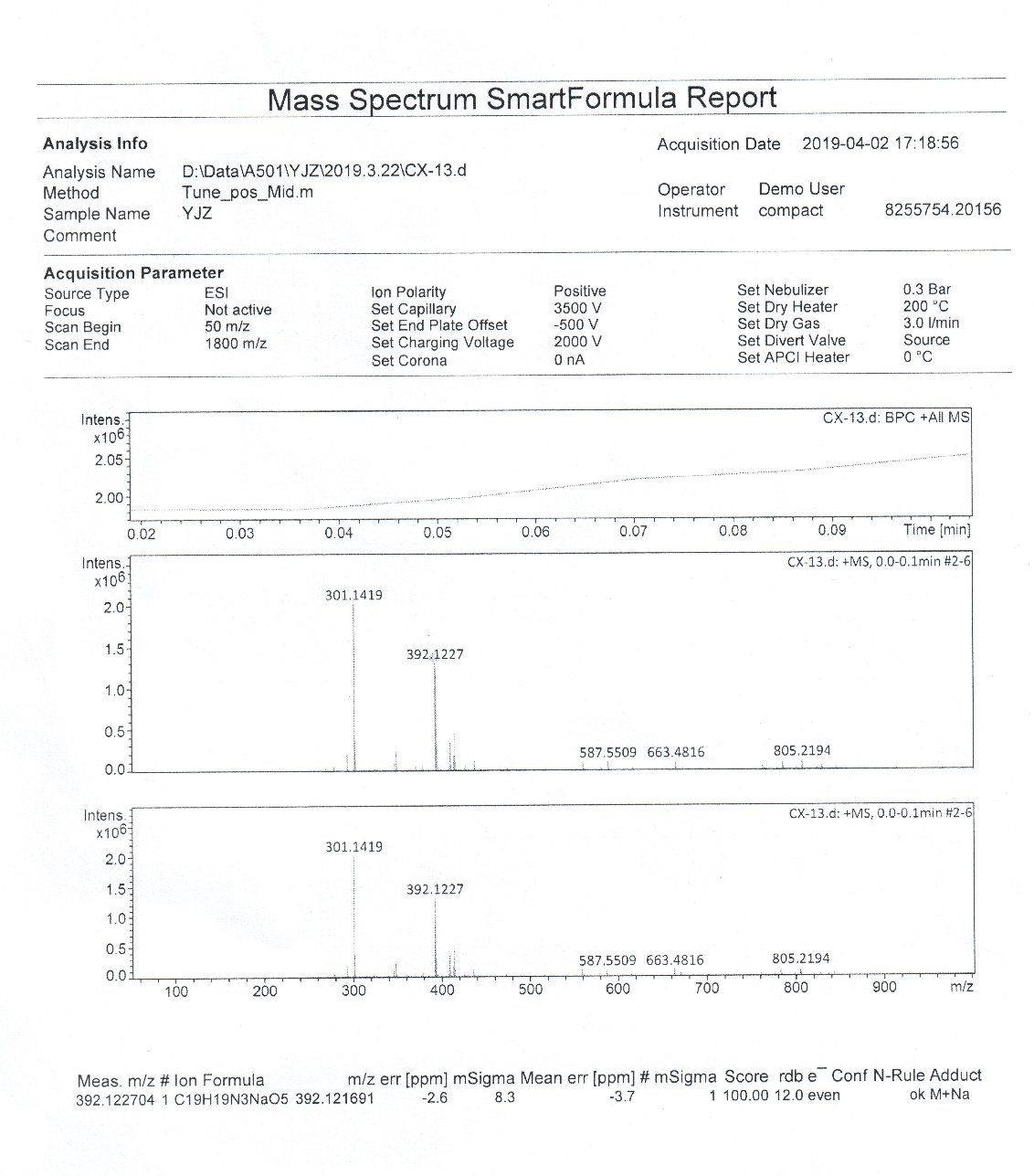


**Supplementary Figure 20.** The ^1^H NMR spectrum of compound **4** in CD_3_OD-*d*_4_ (600 MHz)


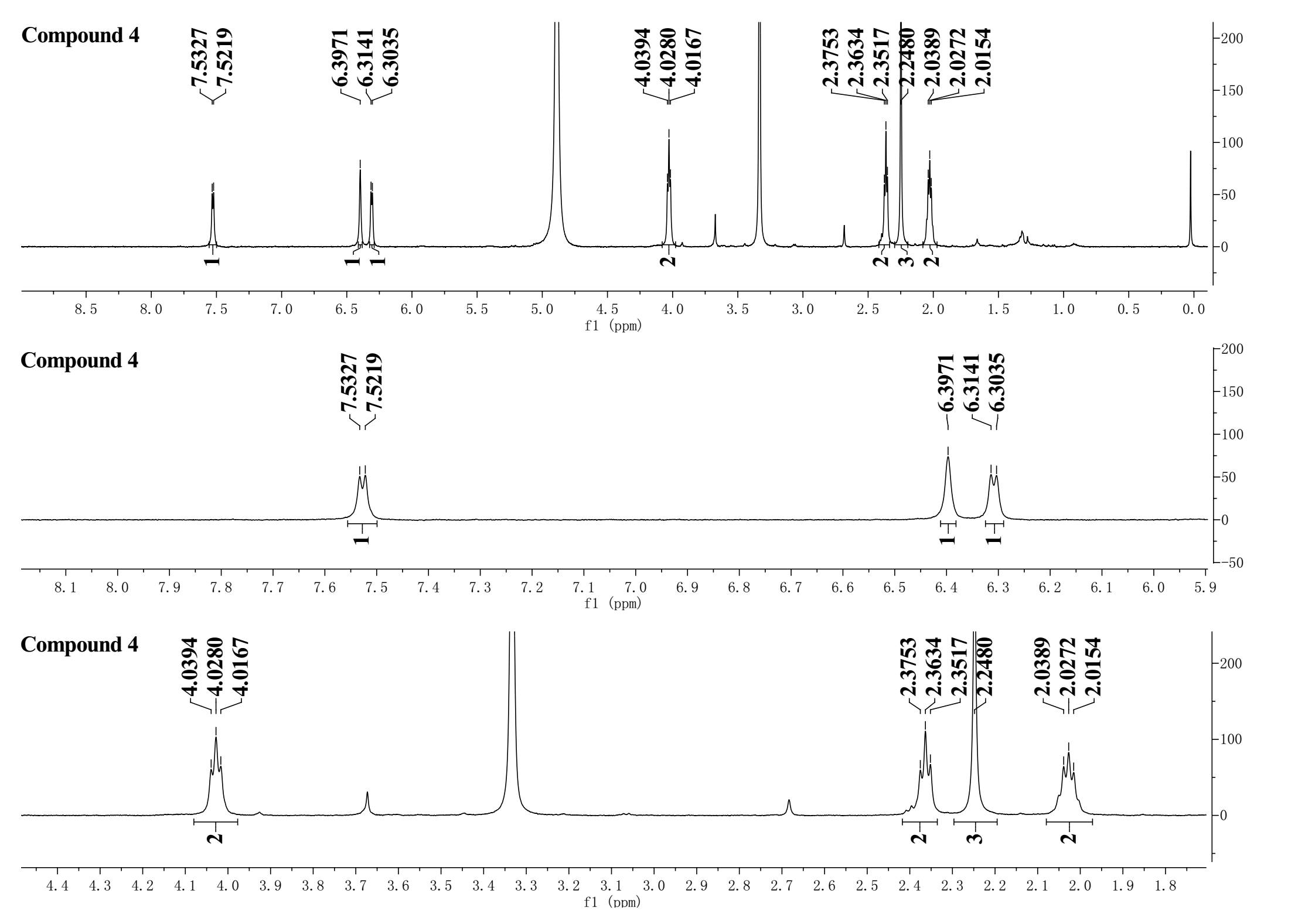


**Supplementary Figure 21.** The DEPTQ spectrum of compound **4** in CD_3_OD-*d*_4_ (125 MHz)


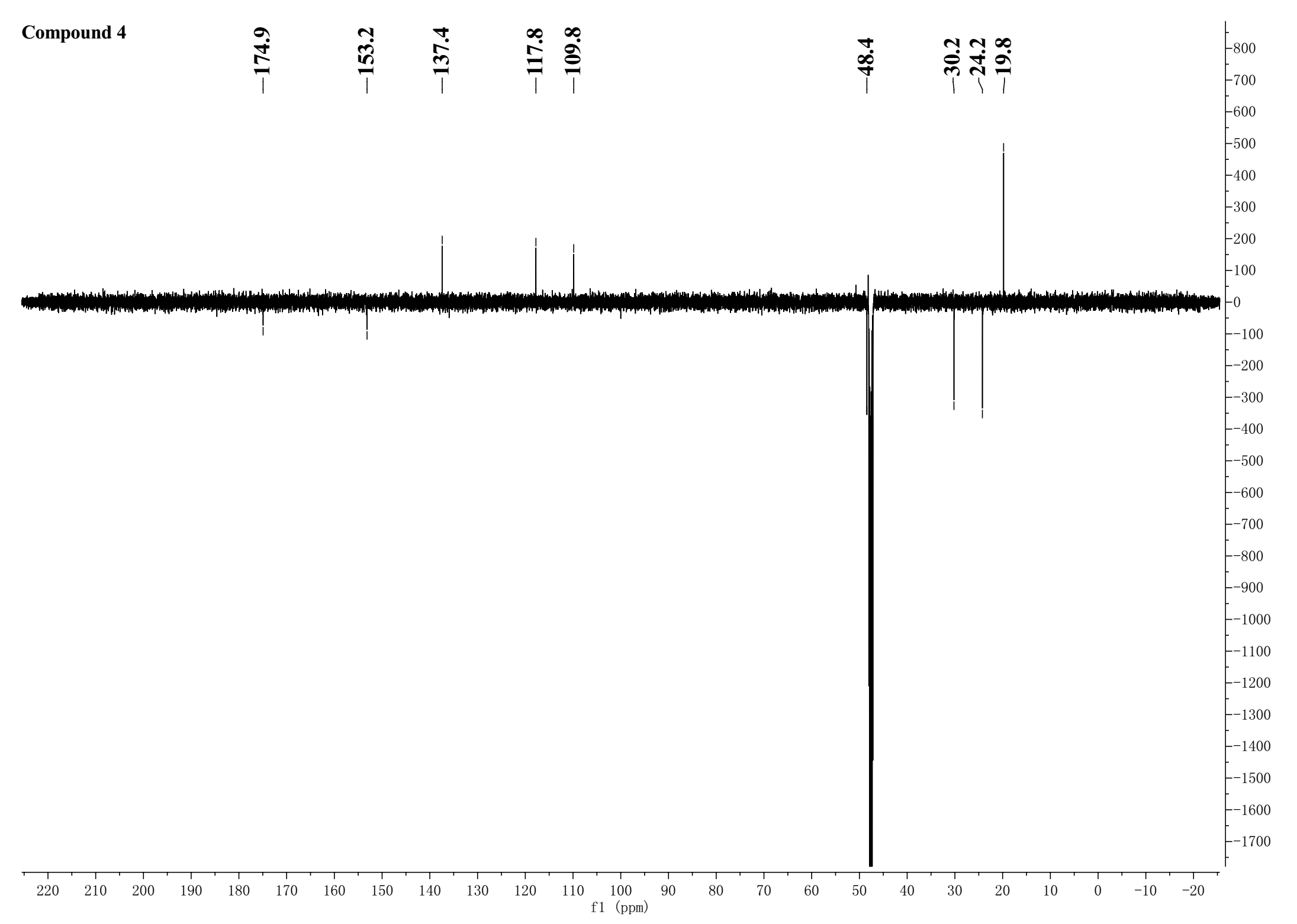


**Supplementary Figure 22.** The HSQC spectrum of compound **4** in CD_3_OD-*d*_4_


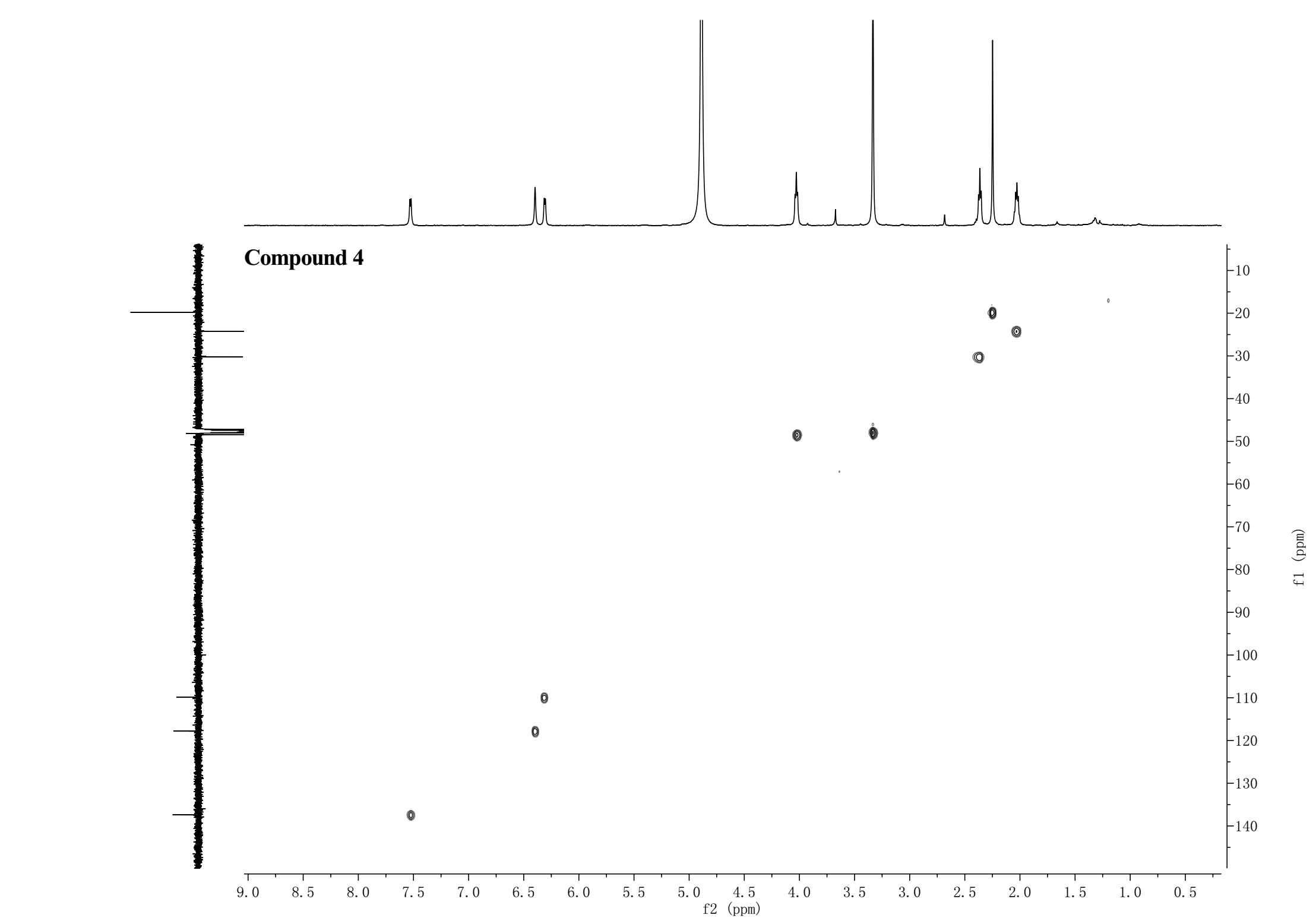


**Supplementary Figure 23.** The ^1^H-^1^H COSY spectrum of compound **4** in CD_3_OD-*d*_4_


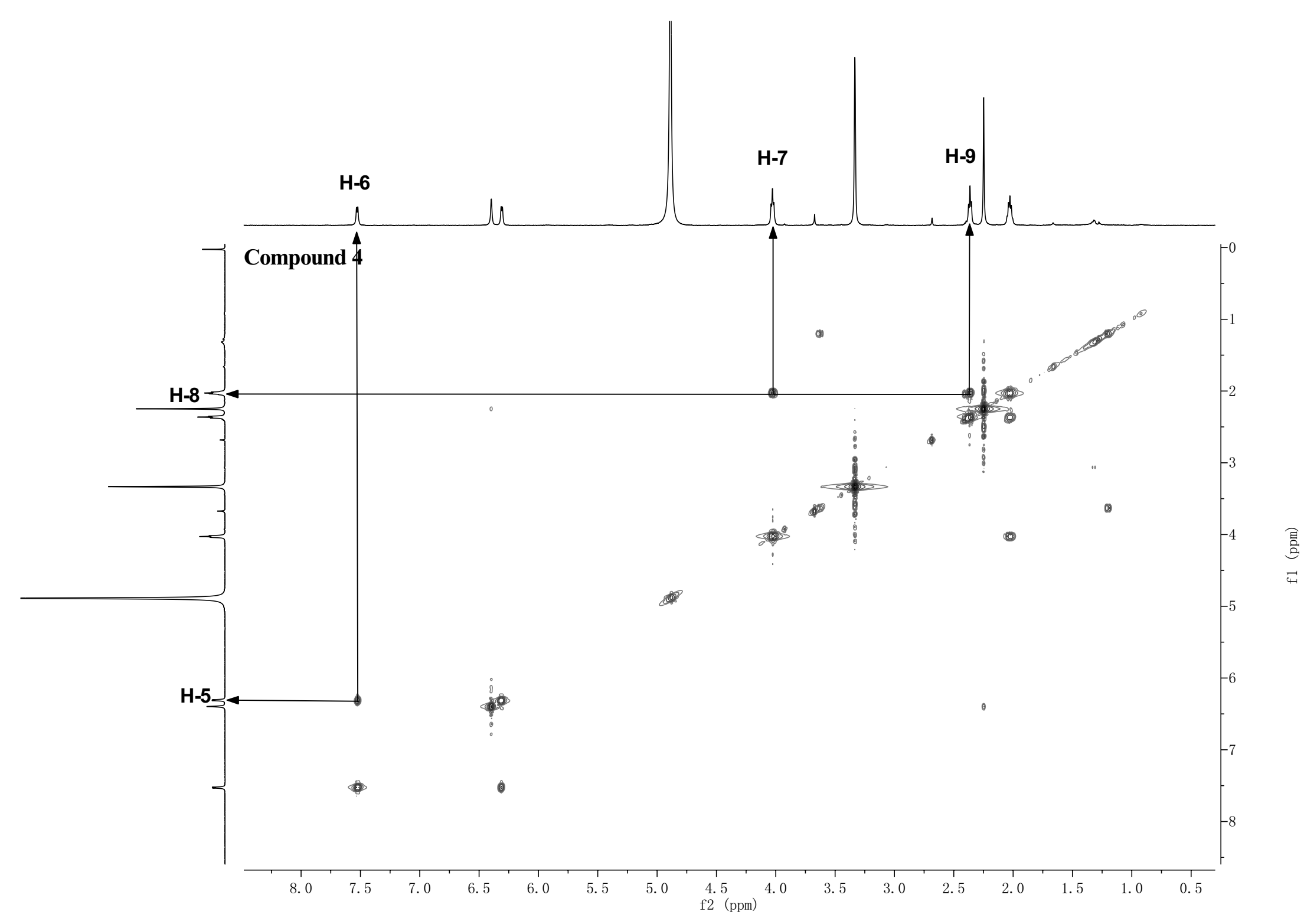


**Supplementary Figure 24.** The HMBC spectrum of compound **4** in CD_3_OD-*d*_4_


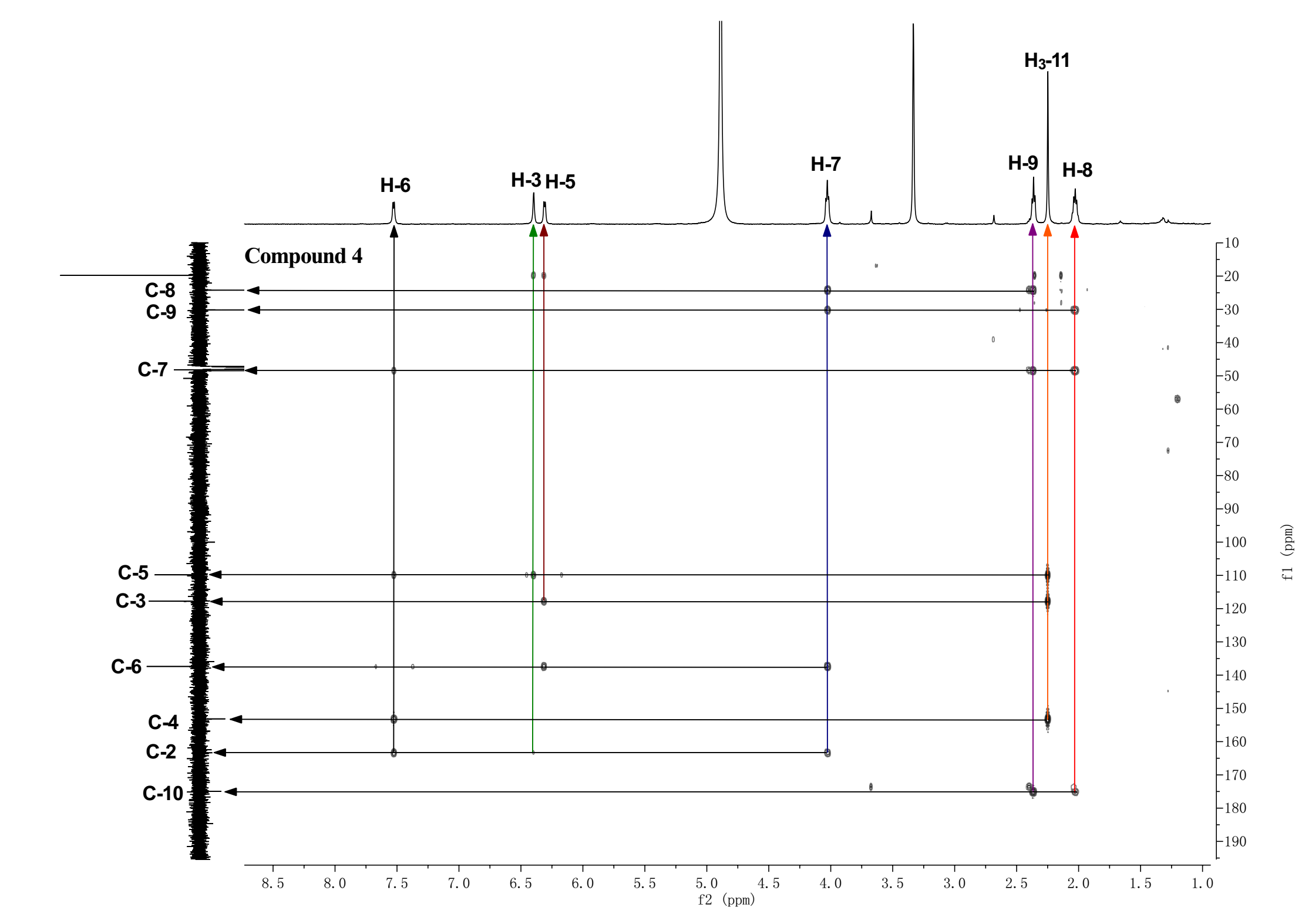

**Supplementary Figure 25.** The HRESIMS spectrum of compound **4**


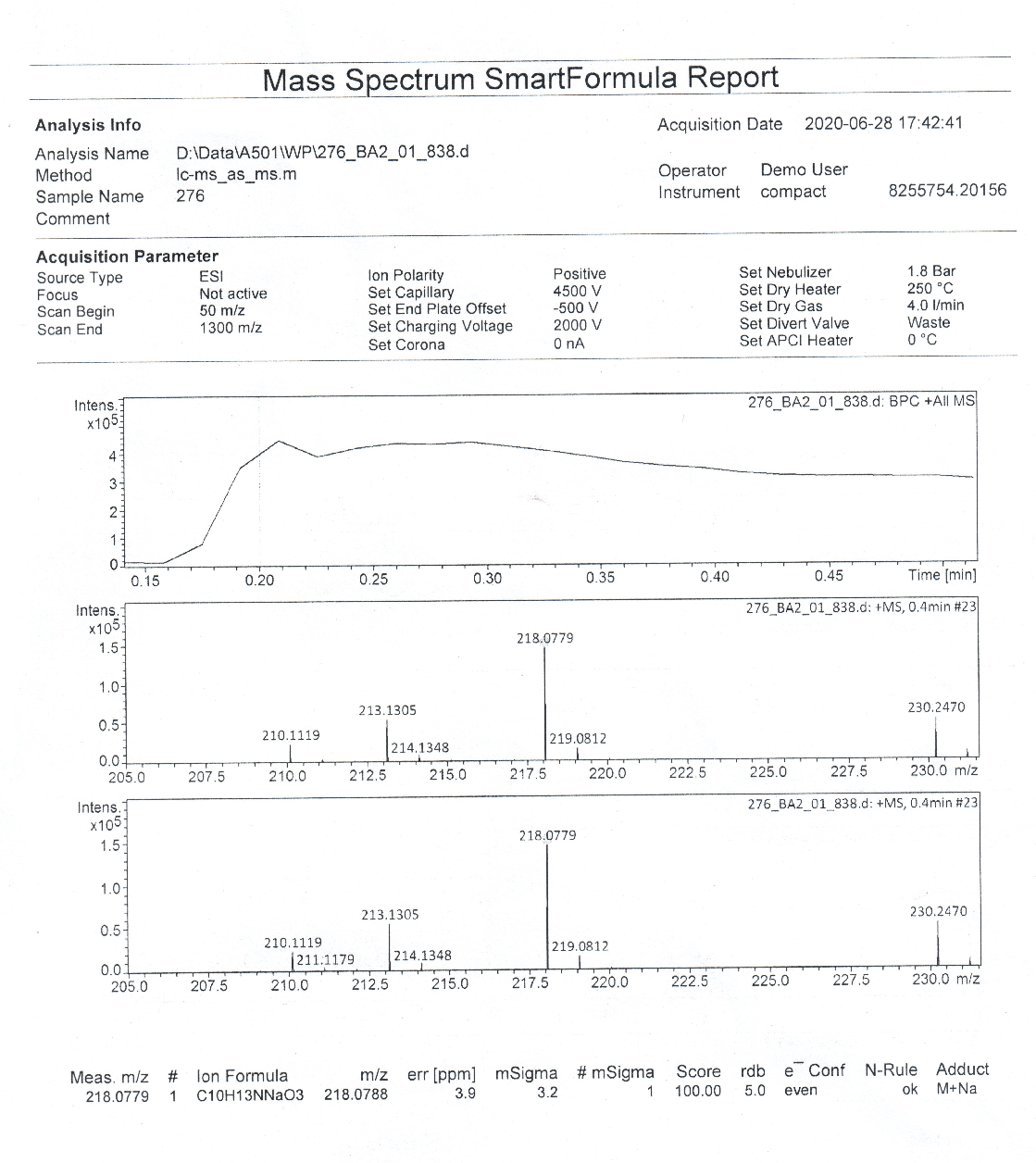


**Supplementary Figure 26**. Marfey’s method applying for compound **3 (**ODS column : Cosmosil-pack, 4.6 × 250 mm, 5 µm, 1 mL/min, Nacalai Tesque; Solvents: (A) water + 0.1% TFA, (B) MeCN; linear gradient: 0 min, 75% A + 25% B; 40 min, 40% A + 60% B; 45 min, 100% B; temperature, 30 °C; flow rate, 1 mL/min; UV detection at λmax 340 nm; FDAA, 15.7 min)**.**


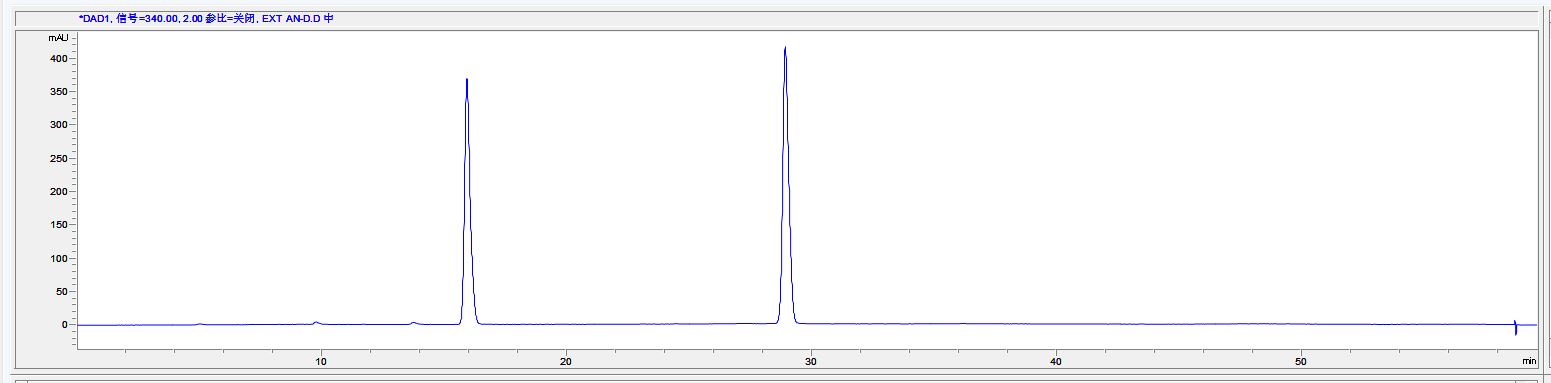

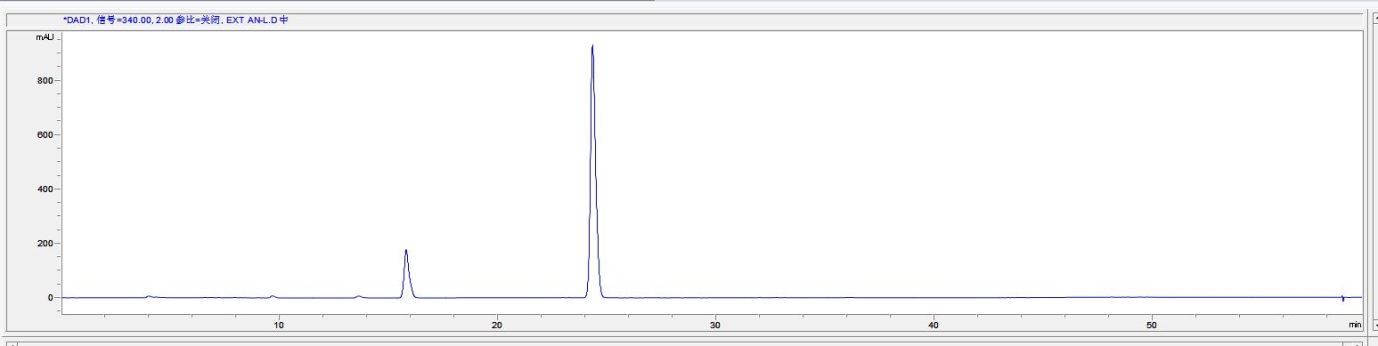

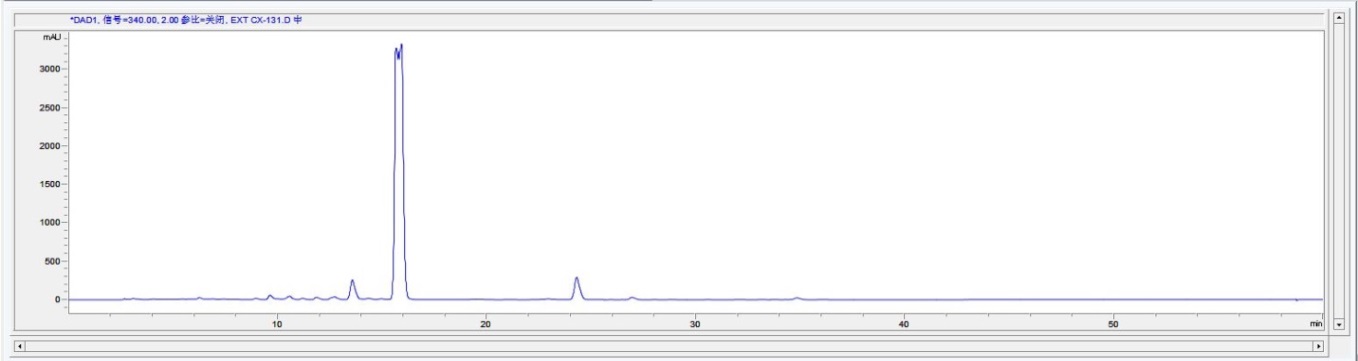


FDAA derivatives of standard L-Ile by ODS column

FDAA derivatives of the acid hydrolysate of **3** by ODS column

FDAA derivatives of standard D-Ile by ODS column


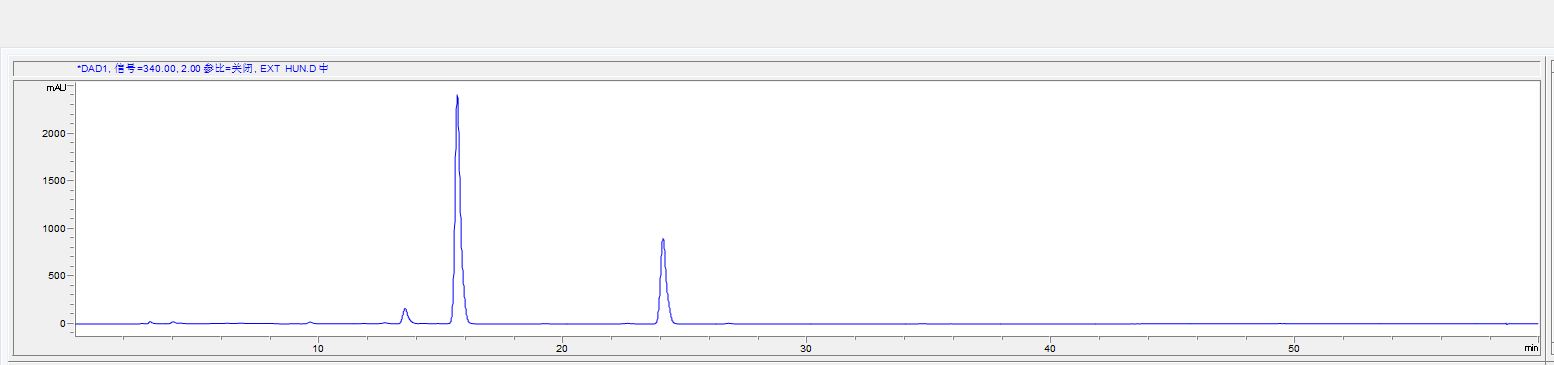


Co-injection of FDAA derivatives of the acid hydrolysates of **3** with standard L-Ile by ODS column

**Supplementary Figure 27**. HPLC analysis of compounds **1**-**3**

Compound **1**: ODS column : Cosmosil-pack, 4.6 × 250 mm, 5 µm, 1 mL/min, Nacalai Tesque; Solvents: (A) water + 0.05% TFA, (B) MeOH; linear gradient: 0 min, 90% A + 10% B; 15 min,

100% B; temperature, 30 °C; UV detection at λmax 262 nm; flow rate, 1; t*_R_* 14.051; Area%: 96.19%.


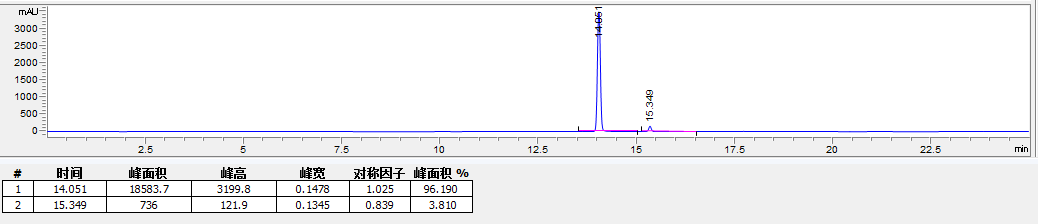


Compound **2**:.ODS column : Cosmosil-pack, 4.6 × 250 mm, 5 µm, 1 mL/min, Nacalai Tesque; Solvents: (A) water + 0.05% TFA, (B) MeOH; linear gradient: 0 min, 90% A + 10% B; 15 min, 100% B; temperature, 30 °C; UV detection at λmax 262 nm; flow rate, 1; t*_R_* 15.335; Area%: 100%.


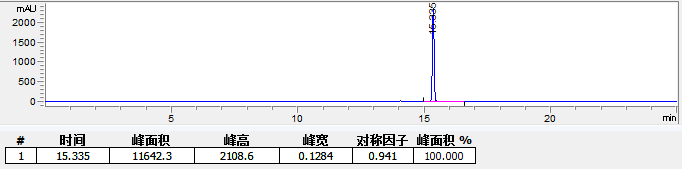


Compound **3**: 5PFP column : Cosmosil-pack, 4.6 × 250 mm, 5 µm, 1 mL/min, Nacalai Tesque; Solvents: (A) water + 0.05% TFA, (B) MeCN; Isocratic system:0 min, 70% A + 30% B; 45 min, 70% A + 30% B; temperature, 30 °C; flow rate, 1; UV detection at λmax 278 nm; t*_R_* 24.888 min; Area%: 96.589%.


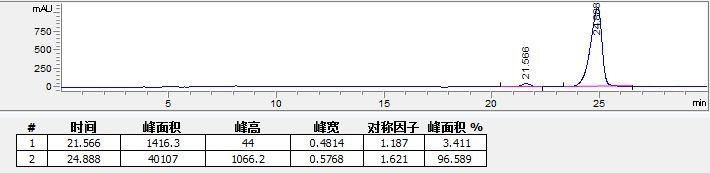


| **No.** | **5**^a^ | | **6** ^a^ | | **7**^b^ | | **8** ^a^ | |
| --- | --- | --- | --- | --- | --- | --- | --- | --- |
|  | ***δ*_C_** | ***δ*_H_, mult.**  (*J* in Hz) | ***δ*_C_** | ***δ*_H_, mult.**  (*J* in Hz) | ***δ*_C_** | ***δ*_H_, mult.**  (*J* in Hz) | ***δ*_C_** | ***δ*_H_, mult.**  (*J* in Hz) |
| 1 | - | - | 175.8, C | - | 172.8, C |  | 175.7, C |  |
| 2 | 135.1,CH | 8.26, br s | 122.7, C | 7.64, br. d, (5.2 ) | 122.8, CH | 7.47, dd, (5.7, 1.5) | 123.0, CH | 7.72, dd, (5.8, 1.5) |
| 3 | 116.6, C | - | 157.1, CH | 6.17, br. d, (5.2 ) | 153.7, CH | 6.21, dd, (5.6, 1.9) | 156.6, CH | 6.19, dd, (6.0, 2.0) |
| 3a | 127.7, C | - | - | - | - | - | - | - |
| 4 | 123.5, CH | 8.2, d, (7.80) | 88.2, CH | 5.08, br. s | 86.1, CH | 4.99, td, (4.8, 1.9) | 88.4 , CH | 5.02, td, (4.9, 1.7) |
| 5 | 124.1, CH | 7.24, d, (7.80) | 71.7, CH | 3.78, m | 71.8, CH | 3.82, m | 72.3, CH | 3.71, m |
| 6 | 125.3, CH | 7.27, d, (8.00 ) | 34.2, CH_2_ | 1.57, overlap | 33.1, CH_2_ | 1.60, overlap | 34.2, CH_2_ | 1.57, m |
| 7 | 113.8, CH | 7.49, d, (8.00 ) | 30.6, CH_2_ | 1.36, overlap | 29.4, CH_2_ | 1.36, overla | 30.7, CH_2_ | 1.38, m |
| 7a | 139.1, CH | - | - | - | - | - | - | - |
| 8 | - | - | 26.8, CH_2_ | 1.36, overlap | 25.5, CH_2_ | 1.36, | 26.8, CH_2_ | 1.38, m |
| 9 | - | - | 26.8, CH_2_ | 1.57, overlap | 25.4, CH_2_ | 1.60, overlap | 26.8, CH_2_ | 1.57, m |
| 10 | - | - | 40.1, CH_2_ | 1.42, m | 39.1, CH_2_ | 1.44, overlap | 40.1, CH_2,_ | 1.43, m |
| 11 | - | **-** | 68.5, CH | 3.70, m | 68.1, CH | 3.77, m | 68.5, CH | 3.71, m |
| 12 | - | - | 23.5, CH_3_ | 1.14, d, (5.6) | 23.6, CH_3_ | 1.19, d, ( 6.0) | 23.5, CH_3_ | 1.14, d, (6.2) |
| 1ˈ | 192.7,C | - | - | - | - | - | - | - |
| 2ˈ | 47.9, CH_2_ | 4.62, s | - | - | - | - | - | - |
| 3ˈ | - | - | - | - | - | - | - | - |
| 4ˈ | 174.2, CH | - | - | - | - | - | - | - |
| 5ˈ | 23.3, CH_3_ | 2.11, s | - | - | - | - | - | - |

**Supplementary Table 1.** ^1^H and ^13^C NMR data for **5-8** (500 and 125 MHz, *δ* in ppm)

^a 1^H and ^13^C NMR Data were measured in CD_3_OD. ^b 1^H and ^13^C NMR Data were measured in CDCl_3_.

**Supplementary Table 2.** Appearance, specific rotation and MS data for **5-8**

| Compounds | Appearance | [α]_D_  (20C, MeOH) | Molecular weghit and Molecular formula |
| --- | --- | --- | --- |
| **5** | white solid | - | 239.0812 [M + Na]^+^  calcd. for C_12_H_12_N_2_O_2_Na: 239.0791 |
| **6** | yellow oil | -127 (*c* 0.2) | 251.1267 [M +Na]^+^  calcd. for C_12_H_20_O_4_Na: 251.1254 |
| **7** | yellow oil | -53 (*c* 0.2) | 251.1239 [M +Na]^+^  calcd. for C_12_H_20_O_4_Na : 251.1254 |
| **8** | yellow oil | +67 (*c* 0.2) | 251.1274 [M +Na]^+^  calcd. for C_12_H_20_O_4_Na : 251.1254 |
